# Identifying physiological and genetic determinants of faba bean transpiration response to evaporative demand

**DOI:** 10.1093/aob/mcad006

**Published:** 2023-01-19

**Authors:** Hend Mandour, Hamid Khazaei, Frederick L Stoddard, Ian C Dodd

**Affiliations:** Lancaster Environment Centre, Lancaster University, Lancaster LA1 4YQ, UK; Genetic Engineering and Biotechnology Research Institute, National Research Centre, Giza, Egypt; Natural Resources Institute Finland (LUKE), Latokartanonkaari 9, 00790 Helsinki, Finland; Department of Agricultural Sciences, Viikki Plant Science Centre and Helsinki Institute of Sustainability Science, PO Box 27 (Latokartanonkaari 5-7), FI-00014 University of Helsinki, Helsinki, Finland; Lancaster Environment Centre, Lancaster University, Lancaster LA1 4YQ, UK

**Keywords:** Faba bean, abiotic stress, transpiration, vapour pressure deficit, hydraulic conductance, genetic control

## Abstract

**Background and Aims:**

Limiting maximum transpiration rate (TR) under high vapour pressure deficit (VPD) works as a water conservation strategy. While some breeding programmes have incorporated this trait into some crops to boost yields in water-limited environments, its underlying physiological mechanisms and genetic regulation remain unknown for faba bean (*Vicia faba*). Thus, we aimed to identify genetic variation in the TR response to VPD in a population of faba bean recombinant inbred lines (RILs) derived from two parental lines with contrasting water use (Mélodie/2 and ILB 938/2).

**Methods:**

Plants were grown in well-watered soil in a climate-controlled glasshouse with diurnally fluctuating VPD and light conditions. Whole plant transpiration was measured in a gas exchange chamber that tightly regulated VPD around the shoot under constant light, while whole-plant hydraulic conductance and its components (root and stem hydraulic conductance) were calculated from dividing TR by water potential gradients measured with a pressure chamber.

**Key Results:**

Although TR of Mélodie/2 increased linearly with VPD, ILB 938/2 limited its TR above 2.0 kPa. Nevertheless, Mélodie/2 had a higher leaf water potential than ILB 938/2 at both low (1.0 kPa) and high (3.2 kPa) VPD. Almost 90 % of the RILs limited their TR at high VPD with a break-point (BP) range of 1.5–3.0 kPa and about 10 % had a linear TR response to VPD. Thirteen genomic regions contributing to minimum and maximum transpiration, and whole-plant and root hydraulic conductance, were identified on chromosomes 1 and 3, while one locus associated with BP transpiration was identified on chromosome 5.

**Conclusions:**

This study provides insight into the physiological and genetic control of transpiration in faba bean and opportunities for marker-assisted selection to improve its performance in water-limited environments.

## INTRODUCTION

Roughly one-third of the world’s arable land is subject to water shortage, which is expected to double by 2050 (Vicente-Serrano *et al*., 2012). Therefore, it is essential to provide farmers with drought-adapted crop varieties to improve yields in water-limited (and well-watered) environments. Among the traits that can ameliorate the effects of water deficits on plant development and performance is limited transpiration rate (TR) under high vapour pressure deficit (VPD), which works as a water conservation strategy to delay the harmful effects of late-season water deficit. Moreover, simulation models that have incorporated this trait into crops such as soybean (*Glycine max*) (Sinclair *et al*., 2010), maize (*Zea mays*) (Messina *et al*., 2015) and lentil (*Lens culinaris*) (Guiguitant *et al*., 2017) revealed that limiting TR above 1–2 kPa (according to the species) resulted in major yield gains under late-season drought environments. Under late-season water deficit, genotypes that limit TR at elevated VPD can potentially use conserved soil water to sustain their physiological performance during grain filling, so they yield more than genotypes that are not expressing the trait (Sinclair *et al*., 2016). Nevertheless, if there is late-season rainfall, the conserved soil water may not be beneficial; thus, genotypes with a limited TR trait would have similar or lower yield than genotypes that do not express the trait (Vadez *et al*., 2014; Sinclair *et al*., 2016). Hence, the yield benefits of the limited-transpiration trait are likely to vary across growing seasons and locations. Thus, limiting TR at high VPD appears to be a promising selection trait, especially in drought-prone areas where crops rely on stored soil moisture.

Selection for limited TR at high VPD in field conditions is always challenged by the requirement of phenotyping the trait in a wide range of environmental conditions (Ghanem *et al*., 2015). Thus, detecting the locations and the effects of genes that regulate limited TR at high VPD is urgently needed using environment-independent DNA markers, especially in drought-sensitive crop species such as faba bean (e.g. Khazaei *et al*., 2014; Muktadir *et al*., 2020). Genomics and transcriptomic approaches now being applied in faba bean open new opportunities for fine mapping and uncovering candidate genes (Khazaei *et al*., 2021). The development of highly saturated and cost-effective second-generation genetic maps has been facilitated by developing DNA markers based on single nucleotide polymorphisms (SNPs) in faba bean (Webb *et al*., 2016; Carrillo-Perdomo *et al*., 2020; Khazaei *et al*., 2021; Gela *et al*., 2022). The SNP markers provide low genotyping cost per data point, high genomic polymorphism, locus specificity in terms of accuracy and reproducibility (e.g. Yan *et al*., 2010; Chongtham *et al*., 2022), simple documentation, co-dominance, a common occurrence amongst elite germplasm (e.g. Cottage *et al*., 2012; Skovbjerg *et al*., 2022), and potential for high-throughput analysis (Wang *et al*., 2021). Recently, a high-density faba bean genotyping array (‘Vfaba_v2’) containing 24 929 polymorphic high-resolution SNP markers located in 15 846 different genes has been developed (O’Sullivan *et al*., 2019; Donal M. O’Sullivan, pers. comm.). Thus, SNP markers are considered powerful tools in genetic mapping, in association studies, assessing genetic diversity and positional cloning in faba bean. In the absence of a published faba bean reference genome, a number of transcriptomes have been reported for faba bean looking for drought-adaptation-related genes (see Alghamdi *et al*., 2018; Khan *et al*., 2019; Wu *et al*., 2020). The large genome of faba bean is currently being assembled, which will further advance faba bean genomics and breeding (Jayakodi *et al*., 2023).

For plants to be able to replace transpirational losses, the soil needs to continuously supply water to the roots; plant transpiration is otherwise restricted under unfavourable conditions such as high VPD. Plants can regulate transpiration at high VPD by matching stomatal and hydraulic conductance to maintain a constant water potential (Attia *et al*., 2015). By decreasing stomatal conductance (*g*_s_) to water vapour, plants minimize water loss and maintain cellular hydration as VPD increases. Despite many reports on transpiration responses to VPD in several crop species, the mechanism(s) of stomatal closure under high VPD remain(s) unclear (Damour *et al*., 2010; Jalakas *et al*., 2021). Maximum transpiration rate and maximum whole-plant hydraulic conductance are positively related (Tsuda and Tyree, 2000) suggesting that hydraulic conductance of different plant organs such as leaves (Sadok and Sinclair, 2010*b*) and roots (Sinclair *et al*., 2014; Sivasakthi *et al*., 2020) can constrain transpiration at high VPDs. However, there is considerable species variation in which organ is perceived to limit TR at high VPD. Limited TR under high VPD was associated with low leaf hydraulic conductance in soybean (Sadok and Sinclair, 2010*b*) and sorghum (*Sorghum bicolor*) (Choudhary *et al*., 2013), while limited root hydraulic conductance was correlated with restricted TR at high VPD in chickpea (*Cicer arietinum*) (Sivasakthi *et al*., 2020). In maize, both leaf and root hydraulic conductance limit TR at high VPD (Choudhary *et al*., 2014). Thus, plant hydraulic conductance seems to play a vital role in regulating the stomatal response to changes in VPD (Sperry *et al*., 2002).

Although restricting transpiration at high VPD can maintain crop yields in dry environments, relatively few studies have sought to determine the genetic basis of this trait (e.g. Schoppach *et al*., 2016; Affortit *et al*., 2022; Tamang *et al.*, 2022) despite the availability of high-throughput phenotyping of the transpiration response to VPD (Ryan *et al*., 2016; Jauregui *et al*., 2018; Kar *et al*., 2020). Screening segregation populations is essential to understand the genetics of limited transpiration at high VPD, particularly in drought-susceptible crops such as faba bean. This research utilized an advanced population of faba bean recombinant inbred lines (RILs) with the following objectives: (1) to identify genotypic variation in TR to VPD, (2) to examine whole-plant hydraulic conductance and its components as a possible regulatory mechanism for limited TR, and (3) to identify genomic regions associated with transpiration response to VPD.

## MATERIALS AND METHODS

### Plant material

The mapping population of faba bean (*Vicia faba* L.) comprised 165 RILs derived from a cross between Mélodie/2 and ILB 938/2 at the *F*_8_ generation (Khazaei *et al*., 2014). Mélodie/2 is an inbred line from INRA (Institut National de la Recherche Agronomique, France) with a relatively high yield and highly efficient use of water, where it maximizes soil moisture capture for transpiration, minimizes water loss by soil evaporation by rapid vegetative growth and reduces non-stomatal transpiration. ILB 938/2 is a selection from an accession originating from the Andean region of Colombia and Ecuador, maintained at ICARDA (International Centre for Agricultural Research in the Dry Areas), with high water use efficiency (WUE, ratio of biomass produced to the rate of transpiration) and relatively low productivity (Khan *et al*., 2007; Khazaei *et al*., 2013, 2018). The parent lines differed in their responses to water deficit. Mélodie/2 had a cooler canopy under well-watered conditions and a much greater increase in canopy temperature under water deficit conditions than ILB 938/2, while *g*_s_ followed the opposite trend. Water deficit induced in potted plants under glasshouse conditions had a three-fold greater effect on biomass production of Mélodie/2 than of ILB 938/2, but biomass in Mélodie/2 under water deficit conditions was the same as in ILB 938/2 under well-watered conditions (Khazaei *et al*., 2014). Thus, ILB 938/2 can maintain higher water status under water deficit conditions as it has high WUE with a relatively low yield. In contrast, Mélodie/2 had better productivity under drought conditions than ILB 938/2, by maintaining water uptake via a well-developed root system (Khazaei *et al*., 2014). Furthermore, the parental lines differed in a wide range of agronomic and morphological characteristics, confirming the wide genetic variation between them and their suitability for genetic mapping and genomic studies. This genetic and geographical divergence made them ideal for building a promising segregating population for successful genetic and trait mapping (Würschum, 2012).

### Growth conditions

A total of 165 RILs from cross Mélodie/2 × ILB 938/2 at the *F*_8_ generation were used to study transpiration, leaf water potential and hydraulic conductance under a range of VPDs in a whole-plant gas exchange chamber (Jauregui *et al*., 2018) between 2019 and 2021. Seeds were chosen randomly and germinated at about 2.5 cm depth in rectangular 2-L pots (12.5 × 10.5 × 21 cm) containing a mixture of commercial John Innes No. 2 substrate (Westland Horticulture Ltd, UK) and silver sand (Royal Horticultural Society, UK) in a ratio of 3 : 1 (v/v). Depending on seed availability, each RIL was represented by three to four plants with seeds planted at different times of the year (Supplementary Data Table S1) in a semi-controlled glasshouse to ensure the replicates were randomly distributed across varying atmospheric conditions in the glasshouse at Lancaster Environment Centre, Lancaster University, UK. Supplementary lighting (high-pressure sodium lamps, Osram Plantastar 600 W, Munich, Germany) maintained the photoperiod at 12 h (0800–2000 h). The light intensity during the photoperiod was 551 ± 3 µmol m^−2^ s^−1^ PPFD (photosynthetic photon flux density) (mean ± s.e., *n* = 3600, comprising 12 h × 300 d) at the pot surface ∼2 m below the lamp. Air temperature and relative humidity in the centre of the glasshouse were recorded hourly with a Hortimax system (HortiMax Ektron III, hortisystems.co.uk). Day/night temperature ranges were 26.1 ± 0.06 and 19.7 ± 0.04 °C (mean ± s.e., *n* = 3600), respectively. Relative humidity day/night ranges were 31 ± 0.2 and 44 ± 0.3 % (mean ± s.e., *n* = 3600), respectively across the entire period of the experiment. These ranges generated a day/night VPD range of 2.32 ± 0.61–1.28 ± 0.60 kPa (mean ± s.e., *n* = 3600), respectively. The plants were grown for ~4 weeks, daily irrigated to the upper limit of pot-drained capacity and fertilized weekly with 0.3 % (w/v) Miracle-Gro All Purpose Plant Food (The Scotts Company Ltd, UK), supplying 20.5 : 3.5 : 3.5 (N : P : K). Homogeneous plants (leaf area = 299 ± 4 cm^2^, mean ± s.e., *n* = 560) were selected based on their developmental stage (seven or eight fully expanded leaves) rather than chronological age. Although leaf area did not differ significantly among RILs, it differed over the year (Supplementary Data Table S1). For example, plants had 35 % higher leaf area in May than those in August (Fig. S1). However, neither genotype nor the genotype × month interaction was significant (Table S1), which was expected since the basic criteria for choosing the plants was leaf number (seven or eight leaves). Plants were assigned to measure transpiration response to VPD in the whole-plant gas exchange between December 2019 and January 2022. Each date assigned a number from 1 (1st January) to 365 (31st December) with no measurements occurring on 28th February.

### Measuring transpiration and hydraulic conductance responses to VPD

TR responses to elevated VPD in the whole-plant gas exchange system were measured on three plants per day, as described by Jauregui *et al.* (2018), from 0900 to 2000 h under six VPD levels within the range ∼1.0–3.5 kPa, with three plants measured per day. Previous experiments with two commercial faba bean cultivars revealed no time of day (morning, afternoon, lateafternoon), or year (July vs. October), effect on the transpiration response to VPD (our unpublished data).

Briefly, the plants were watered to maximum pot-drained capacity and left to drain for about 15 min during which two leaves (the 3rd and 4th from the base of the plant) were covered with aluminium foil to estimate stem water potential (Ψ_stem_) under the lowest and highest VPDs. The plants were then sealed into the chamber and left to acclimate to the chamber lights for about 30 min. Measurements started by increasing chamber relative humidity to its maximum of 70 ± 0.6 % (mean ± s.e., *n* = 560) to generate the lowest VPD while the temperature is stable (25.9 ± 0.09 °C, mean ± s.e., *n* = 560). After CO_2_ and H_2_O exchange had been steady for at least 5 min (steady-state), averaged values were logged every minute for 5 min. The chamber was then opened, and the xylem pressure potential of the aluminium foil-covered leaf (Ψ_stem_) and one fully expanded transpiring leaf (Ψ_leaf_) (15–20 % of total leaf area) across both leaves was measured using a Scholander pressure chamber (Soil Moisture Equipment Corp., Santa Barbara, CA, USA). After closing the chamber again, relative humidity inside the system was further decreased by introducing a mixture of dry and humidified air to the chamber. After the following relative humidity level was achieved, plant gas exchange was allowed to stabilize (typically 30–45 min) and CO_2_ and H_2_O values were logged again. Each plant was exposed to six sequentially decreasing humidity levels (70, 58, 43, 31, 18 and 11 %), achieved by increasing the ratio of dry to humid air approximately corresponding to VPD values of 1.00, 1.41, 1.91, 2.32, 2.75 and 3.20 (±0.02) kPa (mean ± s.e., *n* = 560). Each genotype had at least one plant measured at each time of day. Thus each plant took 3–4 h to quantify its transpiration response to VPD, as demonstrated in Mandour (2022).

Across the entire period of measurements (morning, afternoon and late afternoon), the main driving force for the VPD treatments was variation in the humidity levels established in the whole-plant gas exchange chamber as a result of differing air source humidity, since chamber temperature was stable resulting in VPD ranging from ~1.0 to 3.2 kPa for all RILs. At the highest VPD, Ψ_leaf_ and Ψ_stem_ were determined again. The covered leaves were not included in leaf area calculations for transpiration measurements but were included in total leaf area calculations. After measuring the whole-plant gas exchange response to changing VPD, the plant was removed from the chamber to determine its leaf area using a leaf area meter (Model LI-3100C, Li-Cor, Lincoln, NE, USA). Data were then downloaded from the infra-red gas analyser (LI-6400XT, Li-Cor) comprising records of transpiration in mg H_2_O (which later was normalized to time in minutes and leaf area to obtain transpiration rate, TR), VPD and other physiological parameters. Whole-plant hydraulic conductance (*K*_plant_) and its components, i.e. root hydraulic conductance (*K*_root_) and stem hydraulic conductance (*K*_stem_), were measured only at the lowest and the highest VPD by dividing TR by Ψ_gradient_, as described by Tsuda and Tyree (2000), as follows:


$$\begin{array}{l} K_{plant\text{\textasciitilde{}}\text{-}min}=\;TR_{min}/\;\left(\Psi_{soil}-\Psi_{leaf}\right)at\;the\;lowest\;VPD \end{array}$$

$$\begin{array}{l} K_{plant\text{\textasciitilde{}}\text{-}max}=TR_{max}/\;\left(\Psi_{soil}-\Psi_{leaf}\right)at\;the\;highest\;VPD \end{array}$$

$$\begin{array}{l} K_{root\text{\textasciitilde{}}\text{-}min}=TR_{min}/\;\left(\Psi_{soil}-\Psi_{stem}\right)at\;the\;lowest\;VPD \end{array}$$

$$\begin{array}{l} K_{root\text{\textasciitilde{}}\text{-}max}=TR_{max}/\left(\Psi_{soil}-\Psi_{stem}\right)at\;the\;highest\;VPD \end{array}$$

$$\begin{array}{l} K_{stem\text{\textasciitilde{}}\text{-}min}=TR_{min}/\;\left(\Psi_{stem}-\Psi_{leaf}\right)at\;the\;lowest\;VPD \end{array}$$

$$\begin{array}{l} K_{stem\text{\textasciitilde{}}\text{-}max}=TR_{max}/\left(\Psi_{stem}-\Psi_{leaf}\right)at\;the\;highest\;VPD \end{array}$$

Since the plants were well watered, Ψ_soil_ was considered to equal zero.

### Identifying genomic regions associated with transpiration response to VPD

#### Genotyping.

 Details on genotypic data and linkage map construction of the Mélodie/2 × ILB 938/2 population at the *F*_8_ generation are explained in Gela *et al.* (2022). Briefly, DNA was isolated from 3-d-old germinated embryo axes for 165 RILs as well as the parental lines using the CTAB (cetyl trimethyl ammonium bromide) method, as described previously (Björnsdotter *et al*., 2021). Genotypic data for this population were generated from the Axiom ‘Vfaba_v2’ 60K array (O’Sullivan *et al*., 2019).

*Linkage map construction.* The linkage map was developed by Gela *et al.* (2022). The 35 363 SNP markers were filtered based on polymorphism between parents, segregation distortion using a chi-square (χ^2^) test and missing data. The linkage map was built using both ASMap software (Taylor and Butler, 2017) and MapDisto v.2.1.8 (Heffelfinger *et al*., 2017) with a logarithm of odds (LOD) score of 3.0 and a cut-off recombination value of 0.35. The Kosambi function was used to calculate the map distance in centiMorgans (cM) (Kosambi, 1943). The map included 4089 markers, distributed in six linkage groups corresponding to the six chromosomes of faba bean, and spanned 1229.5 cM (Gela *et al*., 2022).

### QTL mapping of transpiration response to VPD and candidate gene identification

Composite interval mapping (CIM) was used to detect putative quantitative trait locus (QTL) locations of TR_min_, TR_max_, TR_BP_, *K*_plant-min_, *K*_plant-max_, *K*_root-min_ and *K*_root-max_ by Windows QTL Cartographer v.2.5 (Wang *et al*., 2012). The cofactors were determined using the forward and backward method in the standard CIM model with a probability of 0.1 and window size of 5 cM. QTL significance thresholds were determined by 1000 permutations at a significance level of *P* = 0.05. Phenotyping data of 142 RILs with available genotyping data were used for QTL analysis. To determine candidate genes, the sequences of SNP markers that appeared within the QTL intervals were searched using BLASTn (Goodstein *et al*., 2012) in Phytozome v.13 on the reference genome for *Medicago truncatula*. It was notable that no QTLs were identified for the slopes of the TR versus VPD relationships, or for the actual BP values.

### Statistical analysis

Analysis of the TR response to VPD utilized the segmented linear regression model of GraphPad GraphPad Prism 9.3.1 (GraphPad Software Inc., San Diego, CA, 2007), which provides a break-point (BP) value (when the slopes of the fitted regression differ significantly), values of the slopes and their standard errors as well as the regression coefficient. A simple linear regression was applied when the slopes did not differ significantly (Devi *et al*., 2010; Shekoofa *et al*., 2020). Significant genotypic differences (*P *< 0.05) in regression parameters (slopes and BPs), TR, Ψ_leaf_ and hydraulic conductance for the entire population were discriminated using Student’s *t*-test. ANCOVA (for main effects of genotypes, VPD and their interaction) between the parental lines in their TR, Ψ_leaf_ and *K*_plant_ response to VPD and for the effects of planting month on the genotypic differences in leaf area was carried out with the SPSS 27.0 for Windows statistical software package (SPSS, Inc., Cary, NC, USA). Differences between means were considered statistically significant at *P *< 0.05.

## RESULTS

### Physiological responses of the parental lines

**Fig. 1.**
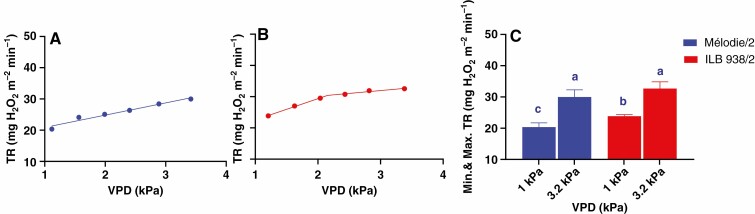
TR response to VPD of Mélodie/2 (A) and ILB 938/2 (B) in the whole-plant gas exchange chamber and the difference between the two genotypes in their minimum (1^st^ column each genotype) and maximum (2^nd^ column each genotype) TR (C). Each point and column represents 5 min of transpiration rate after 15 min of steady-state. Symbols are the mean of four plants per genotype. Error bars were omitted from A and B for clarity, while different letters in C indicate significant (*P* < 0.001) differences according to the *t*-test. Linear (A) and broken-stick (B) regression lines (*P* < 0.01) were fitted in Prism. Mean ± s.e. of regression variables, i.e. slope 1 and *R*^2^ values of Mélodie/2, and BP, slopes and *R*^2^ values of ILB 938/2 are shown on the top of panels A and B.

The parental genotypes differed in their TR response to VPD, as indicated by a significant genotype × VPD interaction (*P* = 0.036, Table 1). While TR of Mélodie/2 increased linearly over the range of VPDs tested (Fig. 1A), that of ILB 938/2 was well characterized by the two-segmental analysis (Fig. 1B). Its TR increased linearly with increasing VPD to reach 30.45 ± 2.35 mg H_2_O m^−2^ min^−1^ at 2.12 ± 0.04 kPa, which represented a BP. Thereafter, TR was relatively stable despite increases in VPD, reaching 32.53 ± 2.25 mg H_2_O m^−2^ min^−1^ at 3.20 ± 0.07 kPa. Although the minimum TR of ILB 938/2 was 17 % higher than that of Mélodie/2 at the lowest VPD, they did not differ significantly in their maximum TR (Fig. 1C). Thus, ILB 938/2 restricted its transpiration at high VPD.

**Table 1.** *P*-values from ANCOVA describing the difference between Mélodie/2 and ILB 938/2 in transpiration rate (TR), leaf water potential (Ψ_leaf_), stem water potential (Ψ_stem_), whole-plant hydraulic conductance (*K*_plant_), root hydraulic conductance (*K*_root_) and stem hydraulic conductance (*K*_stem_) responses to VPD. Significant values are in italics.

Trait	Genotype	VPD	Genotype × VPD
TR	0.15	<*0.001*	*0.036*
Ψ_leaf_	*0.03*	<*0.001*	0.94
Ψ_stem_	*0.002*	<*0.001*	0.41
*K*_plant_	0.90	*0.047*	0.96
*K*_root_	0.76	0.10	0.79
*K*_stem_	0.81	0.06	0.62

**Fig. 2.**
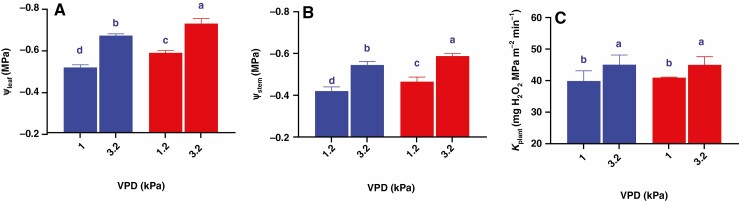
Changes in Ψ_leaf_ (A), Ψ_stem_ (B) and *K*_plant_ (C) in Mélodie/2 (blue columns) and ILB 938/2 (red columns) from the lowest (1 kPa) to the highest (3.2 kPa) VPD. Bars show the mean ± s.e. of four plants of each parental line, with different letters above the bars indicating significant (*P* < 0.05) differences according to the *t*-test.

Leaf water potential (Ψ_leaf_) and stem water potential (Ψ_stem_) responded similarly to VPD in the two genotypes (no significant genotype × VPD interactions – Table 1), with high VPD decreasing Ψ_leaf_ by 0.15 MPa (averaged across genotypes). However, Ψ_leaf_ of Mélodie/2 was significantly higher than that of ILB 938/2 at the lowest VPD (by 14 %) and the highest VPD (by 9 %), respectively (Fig. 2A). Stem water potential (Ψ_stem_) exceeded Ψ_leaf_ by ~25 %, with high VPD decreasing Ψ_stem_ by 0.12 MPa (averaged across genotypes) at the two tested VPDs and decreased by 30 and 26 % at the highest VPD in Mélodie/2 and ILB 938/2, respectively (Fig. 2B). Whole-plant hydraulic conductance (*K*_plant_) did not differ between genotypes but was increased similarly by 12 % (averaged across genotypes) at the highest VPD, as indicated by no significant genotype × VPD interaction (Table 1; Fig. 2B). Thus, lower Ψ_leaf_ and Ψ_stem_ of ILB 938/2 (irrespective of VPD) could not be attributed to impaired *K*_plant_.

**Fig. 3.**
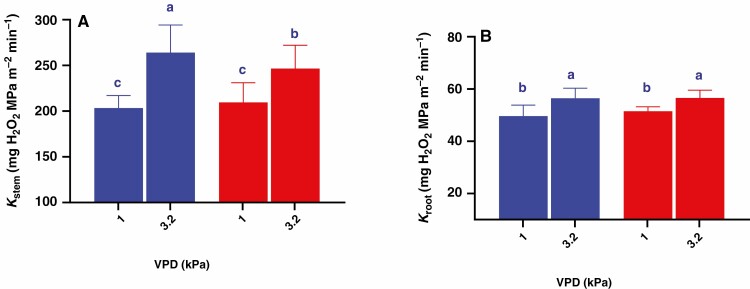
Changes in *K*_stem_ (A) and *K*_root_ (B) in Mélodie/2 (blue columns) and ILB 938/2 (red columns) from the lowest to the highest VPD. Bars show the mean ± s.e. of four plants of each parental line, with different letters above the bars indicating significant (*P* < 0.05) differences according to the *t*-test.

Stem hydraulic conductance (*K*_stem_) and root hydraulic conductance (*K*_root_) responded similarly to VPD in the two genotypes (no significant genotype × VPD interactions, Table 1), even if *K*_stem_ was 4- and 4.5-fold higher than *K*_root_ in Mélodie/2 and ILB 938/2 respectively at the two tested VPDs (Fig. 3A and B). Whereas both genotypes had similar *K*_stem_ at the lowest VPD, *K*_stem_ of Mélodie/2 was 8 % higher than that of ILB 938/2 at the highest VPD (Fig. 3A). In contrast, *K*_root_ did not differ significantly between the genotypes at any VPD (Fig. 3B). While *K*_stem_ of Mélodie/2 increased by 30 % as VPD increased, ILB 938/2 increased its *K*_stem_ by 18 % at the highest VPD (Fig. 3A). Overall, the component hydraulic conductances (*K*_stem_ and *K*_root_) did not show genotypic differences (as with *K*_plant_) but increased at the highest VPD.

### Genotypic variation in TR response to VPD in the RILs

**Fig. 4.**
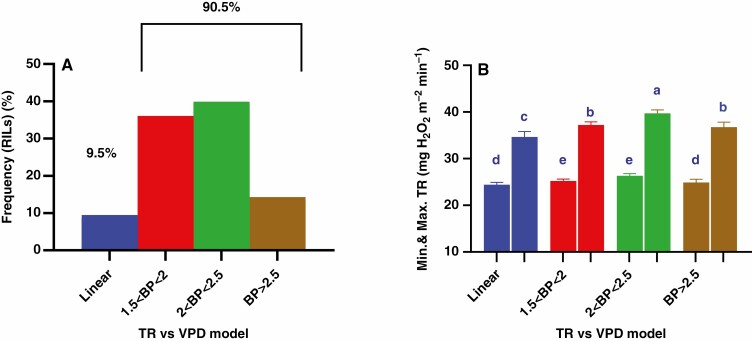
Frequency distribution ( %) of the TR response models to VPD of 165 RILs derived from Mélodie/2 and ILB 938/2 (A) and the difference between the groups in their minimum TR (1^st^ column in each group) and maximum (2^nd^ column in each group) TR (B). Bars show mean ± s.e. of TR of the genotypes in each group (*n* = 585), with different letters above the bars indicating significant (*P* < 0.05) differences according to the *t*-test.

As the parental lines differed, there was considerable variability in the TR response to VPD amongst the progeny lines. Segmented regression analysis identified a significant BP in more than 90 % of the population (150 RILs) ranging from 1.5 to 3.0 kPa, while only 15 RILs had a linear TR model (Fig. 4A). The RILs with a segmented TR response were divided into three sub-groups based on the BP value as follows: (1) 1.5 < BP < 2.0 (61 RILs), 2) 2.0 < BP < 2.5 (65 RILs) and BP > 2.5 (24 RILs). At the lowest VPD, the groups of RILs (linear and segmented TR) differed slightly in their TR, with TR of the groups 1.5 < BP < 2.0 and 2.0 < BP < 2.5 exceeding that of the linear group and BP > 2.5 groups by 5 %, averaging 25.0 ± 0.73 mg H_2_O m^−2^ min^−1^. The highest VPD increased TR by 42–51 %, with the group having a BP between 2.0 and 2.5 kPa having the highest TR of 39.72 ± 1.45 mg H_2_O m^−2^ min^−1^, the group showing a linear response to TR having the lowest TR of 34.36 ± 1.24 mg H_2_O m^−2^ min^−1^ and the other two groups showing an intermediate response. Thus, the different groups varied in their maximum TR (Fig. 4B).

**Fig. 5.**
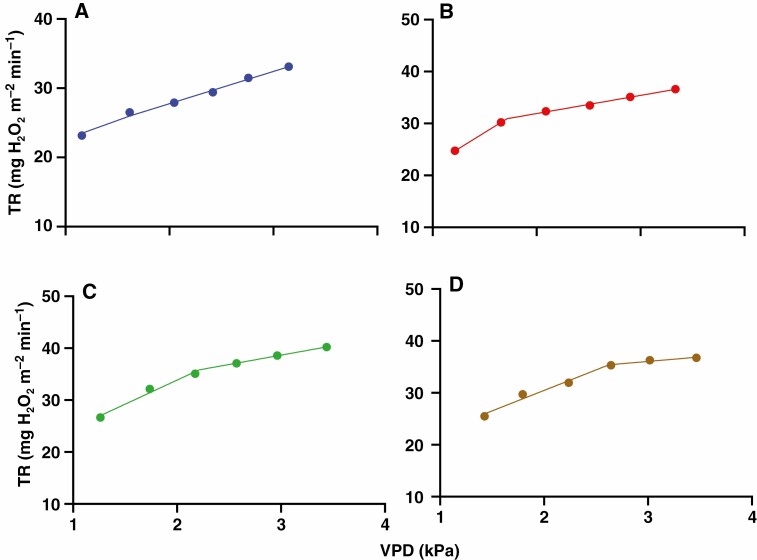
TR response to VPD of the linear TR group (A), and the three segmented TR models, i.e 1.5 < BP < 2 kPa (B), 2 < BP < 2.5 kPa (C) and BP > 2.5 kPa (D). Data are presented as the mean TR of genotypes in each group. Linear (A) and broken-stick (B–D) regression lines (*P* < 0.01) were fitted in Prism. Mean ± s.e. of regression variables, i.e. BP, slopes, R^2^ and *P*-value, are represented on the top of each panel. Symbols are the mean of four plants per genotype, comprising a, b, c and d plants in panels A, B, C and D respectively with error bars omitted for clarity.

In the group where TR was linearly related to VPD (*R*^2^ averaged 0.95 ± 0.01), the slope averaged 4.86 ± 0.35 mg H_2_O m^−2^ min^−1^ kPa^−1^, similar to the parental line Mélodie/2 (4.92 ± 0.94 mg H_2_O m^−2^ min^−1^ kPa^−1^) (Fig. 5A). For the 61 RILs with a low BP (1.5 < BP < 2.0), slope 1 (below the BP) was substantially (28–47 %) greater than in the other groups. For this group, slope 2 (above the BP) was 0.5- to 2.5-fold greater than in the other groups (Fig. 5B–D; Table 2). In genotypes that restricted their transpiration at lower VPDs (lower BP), their transpiration was less constrained by increasing VPDs.

**Table 2.** Differences between the parental lines and the four groups of RILs in their slope 1, break point (BP) and slope 2 of the transpiration response to VPD. Data are the mean ± s.e. of the individual parental lines, or the number of RILs (165).

Parental line/group	Slope 1	BP	Slope 2
Mélodie/2	4.92 ± 0.94^d^	NA	NA
ILB 938/2	6.81 ± 1.44^c^	2.15 ± 0.09^b^	1.82 ± 0.89^b^
Linear	4.86 ± 0.35^d^	NA	NA
1.5 < BP < 2.0	11.93 ± 0.54^a^	1.75 ± 0.02^c^	3.52 ± 0.16^a^
2.0 < BP < 2.5	9.36 ± 0.36^b^	2.24 ± 0.02^b^	2.93 ± 0.18^a^

Different letters indicate significant (*P* < 0.05) differences according to the *t*-test. NA, not applicable.

**Fig. 6.**
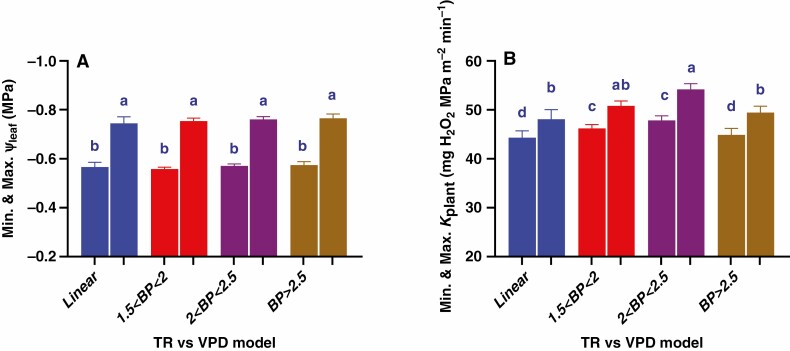
Differences between the four TR models of 165 RILs in their Ψ_leaf_ (A) and *K*_plant_ (B) at the lowest (1^st^ column in each group) and the highest (2^nd^ column in each group) VPD levels. Data are presented as mean ± s.e. of three or four plants of each RIL comprising a, b, c and d plants for the linear, 1.5 < BP < 2, 2 < BP < 2.5 and BP < 2.5 groups respectively, with different letters above the bars indicating significant (*P* < 0.05) differences according to the *t*-test.

Similar to the parental lines, leaf water potential (Ψ_leaf_) did not differ between the four groups at the two tested VPDs, averaging −0.567 ± 0.012 MPa at the lowest VPD and −0.756 ± 0.016 MPa at the highest VPD (Fig. 6A). Whole-plant hydraulic conductance (*K*_plant_) differed significantly across the four groups, with greater values in the 2.0 < BP < 2.5 kPa group, i.e 47.85 ± 0.93and 54.19 ± 1.15 mg H_2_O m^−2^ min^−1^ kPa^−1^ at the lowest and the highest VPD, respectively. These values were 6-12 % higher than *K*_plant_ in the other groups and 10–24 % higher than the parental lines (Fig. 6B). Tripling the VPD increased *K*_plant_ by 9–14 % across the four groups with the highest increase in the 2.0 < BP < 2.5 group and the lowest in the linear group, causing significant differences between the four groups at the two tested VPDs.

**Fig. 7.**
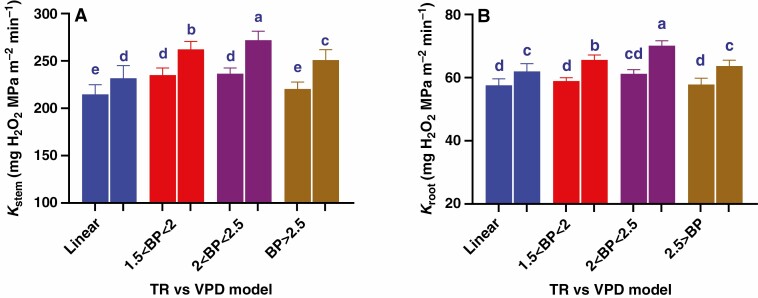
Differences between the four TR models of 165 RILs in their *K*_stem_ (A) and *K*_root_ (B) at the lowest (1^st^ column in each group) and the highest (2^nd^ column in each group) VPD levels. Data are presented as mean ± s.e. of three or four plants of each RIL comprising a, b, c and d plants for the linear, 1.5 < BP < 2, 2 < BP < 2.5 and BP < 2.5 groups respectively, with different letters above the bars indicating significant (*P* < 0.05) differences according to *t*-test.

Across the entire population, *K*_stem_ and *K*_root_ differed significantly between the groups at the two tested VPDs with about three-fold lower *K*_root_ values than *K*_stem_ (Fig. 7A and B). The highest VPD increased *K*_stem_ and *K*_root_ by 8–15 % across the four groups, with the greatest increase in the 2.0 < BP < 2.5 group and the lowest within the linear one (Fig. 7A and B). At the lowest VPD, *K*_stem_ of the 1.5 < BP < 2.0 and 2.0 < BP < 2.5 groups exceeded the other groups by 8 % (averaged across the groups) while at the highest VPD, *K*_stem_ differed significantly between all four groups with the highest values recorded for the 2.0 < BP < 2.5 group (Fig. 7A). Similar differences in *K*_root_ were detected at the highest VPD but were absent at the lowest VPD (Fig. 7B). Taken together, the 2.0 < BP < 2.5 group sustained higher transpiration rates at high VPD, associated with its higher *K*_plant_ and its components.

### QTL analysis and candidate gene identification

**Fig. 8.**
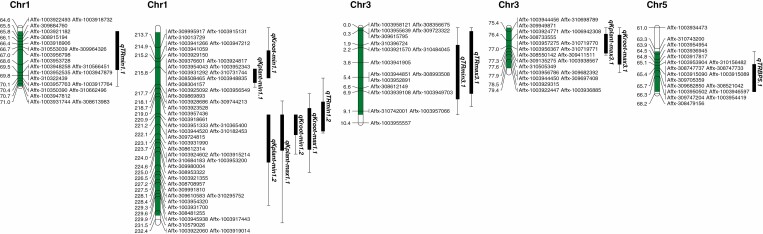
Location of QTLs on chromosomes 1, 3 and 5 using 142 RILs derived from cross Mélodie/2 × ILB 938/2. The QTL interval regions are shown with a green bar. QTLs are represented by boxes extended by lines representing the LOD-1 and LOD-2 confidence intervals. Only portions of the linkage map including the QTL positions are displayed. Full-length chromosomes are presented in Gela *et al.* (2022).

Thirteen QTLs were identified in total: three for the TR_min_ on chromosomes 1 and 3, one for TR_max_ on chromosome 3, one for TR_BP_ on chromosome 5, two for *K*_plant-min_ on chromosome 1, two for *K*_plant-max_ on chromosomes 1 and 3, two for *K*_root-min_ on chromosome 1, and two for *K*_root-max_ on chromosomes 1 and 3 (Table 3; Fig. 8; Supplementary Data Fig. S2). The QTLs qTR_min_1.1, qTR_min_1.2 and qTR_min_3.1 each accounted for over 9 % of the phenotypic variance explained (PVE). qTR_min_1.1 and qTR_min_3.1 showed a negative additive effect, suggesting that the positive allele came from Mélodie/2, whereas qTR_min_1.2 showed a positive additive effect, indicating that its positive allele came from ILB 938/2. The QTLs qK_plant-min_1.1, qK_plant-min_1.2, qK_plant-max_1.1 and qK_plant-max_3.1 accounted for 12, 13, 10 and 10 % of the PVE, respectively. All of these QTLs showed positive additive effects, so alleles for higher values were probably associated with ILB 938/2. QTLs qK_root-min_1.1, qK_root-min_1.2, qK_root-max_1.1 and qK_root-max_3.1, explained 11, 9, 10 and 11 % of the PVE, respectively. The positive additive effect suggests that its positive allele came from ILB 938/2. Similarly, qTR_BP_ showed about 11 % of variation with a positive additive effect. QTLs governing TR_min_, *K*_plant-min_, *K*_plant-max_, *K*_root-min_ and *K*_root-max_ were co-located on the same region on chromosome 1. Likewise, QTLs for TR_min_ and TR_max_ and QTLs for *K*_plant-max_ and *K*_root-max_ were co-located on chromosome 3 (Table 3; Fig. 8). Several candidate genes were also identified in the corresponding regions that may play a role in traits related to transpiration efficiency and plant–water relationships (Supplementary Data Table S2).

**Table 3.** QTL information for minimum and maximum transpiration rate (TR_min_ and TR_max_), whole-plant (*K*_plant-min_ and *K*_plant-max_) and root hydraulic conductance (*K*_root-min_ and *K*_root-max_), and break-point transpiration (TR_BP_) traits in Mélodie/2 × ILB 938/2 RIL population at *F*_8_F8.

QTL	Chromosome	Peak (cM)	QTL interval	LOD	*R* ^2^ (%)^a^	Add^b^
qTR_min_1.1	1	67.01	65.0–69.0	3.59	9.13	−1.02
qTR_min_1.2	1	222.01	217.0–219.0	3.41	9.40	1.18
qK_plant-min_1.1	1	217.01	216.0–218.0	4.64	11.58	2.34
qK_plant-min_1.2	1	222.01	221.0–226.0	5.07	12.54	2.40
qK_plant-max_1.1	1	222.01	221.0–224.90	4.73	11.80	2.90
qK_root-max_1.1	1	222.01	220.30–224.80	3.57	9.90	3.50
qK_root-min_1.1	1	215.0	214.0–217.0	4.51	11.10	3.11
qK_root-min_1.2	1	222.01	221.0–225.30	3.51	8.70	2.80
qTR_min_3.1	3	5.01	1.90–9.50	3.35	9.36	−1.12
qTR_max_3.1	3	5.01	0.40–8.60	4.02	10.80	−2.03
qK_plant-max_3.1	3	77.01	75.70–78.0	4.05	10.60	2.79
qK_root-max_3.1	3	77.01	75.8–79.0	4.04	10.88	3.73
qTR_BP_5.1	5	66.0	63.0–68.0	4.01	10.80	1.55

^a^ *R* ^2^, percentage of phenotypic variance explained by QTL.

^b^Additive genetic effect.

## DISCUSSION

This study identified genomic regions governing the TR and hydraulic conductance response to evaporative demand in a bi-parental mapping population of faba bean. Although both parental lines (Mélodie/2 and ILB 938/2) were considered drought-adapted (Abdelmula *et al*., 1999; Khan *et al*., 2007; Khazaei *et al*., 2014), they differed significantly in their TR response to VPD, where TR of Mélodie/2 increased linearly as VPD increased while ILB 938/2 restricted its TR once the VPD exceeded 2.1 kPa. Nevertheless, both genotypes achieved the same maximum TR. However, this transpirational restriction of ILB 938/2 did not prevent it from having a lower Ψ_leaf_ than Mélodie/2 irrespective of VPD (Fig. 2A), and Ψ_leaf_ of both genotypes declined similarly as VPD increased (Table 1). Similarly, whole-plant hydraulic conductance (*K*_plant_) did not differ between genotypes irrespective of VPD (Fig. 2C), disproving our hypothesis that limited *K*_plant_ restricted transpiration. Although stem hydraulic conductance of ILB 938/2 was less than that of Mélodie/2 at high VPD (Fig. 3A), this difference was insufficient to significantly affect *K*_plant_, and both genotypes achieved a similar maximum TR (Fig. 1C). Limited agreement between variation in hydraulic conductance and transpiration responses to VPD suggests that alternative (non-hydraulic) mechanisms may restrict transpiration at high VPD, with foliar abscisic acid (ABA) accumulation possible (Kholová *et al*., 2010; McAdam *et al*., 2016). Indeed, ABA-deficient mutants fail to show stomatal closure at high VPD (McAdam *et al*., 2015, 2016).

Since transpiration of the parental lines differed in response to VPD, it was likely that RILs derived from these lines would also show differences. Only 15 RILs (10 %) were represented by a single linear regression over the entire range of VPD that matched Mélodie/2, and 65 RILs (40 %) exhibited a segment TR response with a 2.0 < BP < 2.5 that matched ILB 938/2. Interestingly, half of the population revealed a segmented TR response with a BP lower (1.5–2.0 kPa, 61 RILs) and higher (>2.5 kPa, 24 RILs) than the BP of ILB 938/2. The observed stability or even the slightly higher TR after the BP is beneficial for improving crop performance under mild abiotic stress as an alternative to stomatal closure under severe stress conditions (Collins *et al*., 2008). The low BP of 61 RILs suggests that whole-plant hydraulic conductance may be restricted more than that of either parent. Since the TR response to VPD in almost half of the RILs differed from the parents, this trait has a complex inheritance consistent with a previous study in soybean (Sadok and Sinclair, 2009). None of the soybean genotypes that had a segmented TR genotype in their pedigree expressed the segmented TR trait, indicating that the trait(s) responsible for this response is either recessive or depends on a combination of alleles.

A better understanding of the genetic basis of this variability requires QTL analysis of the TR response to VPD. Theoretically, genotypes with a low BP are probably better suited to a dry environment than those with a high BP, as they restrict transpirational depletion of soil moisture reserves. However, genotypes with a low BP may not necessarily be the most water-conserving if their transpiration increases more rapidly at VPDs below the BP (higher slope 1 values). Paradoxically, those genotypes with a moderate BP (2.0–2.5 kPa) sustained a higher maximum transpiration rate than those with higher and lower BPs and the linear group (Fig. 4B), indicating the VPD of the BP (or its occurrence) did not coincide with the lowest maximum TR. Instead, genotypes that did not restrict transpiration as VPD increased actually had the lowest maximum TR (Weatherley, 1982; Else *et al*., 1995; Steudle and Peterson, 1998) consistent with the response of faba bean (Fig. 4B). These findings suggest considerable diversity in the relationships between transpiration responses to evaporative demand and the existence of any hydraulic restrictions.

In the mapping population used in this study, ψ_leaf_ decreased by 30–35 % at the highest VPD (Fig. 6A), although independently of whether the genotypes restricted transpiration at high VPD. Possibly the stomata directly sense high VPD independently of the bulk leaf water potential and close before the leaf experiences water shortage. In control terms, this may be regarded as a feed-forward response of the stomata to high evaporative demand (Bunce, 1997; Dewar, 2002; Buckley, 2005).

By measuring components of hydraulic conductance *in planta*, root hydraulic conductance was identified as the most limiting to whole-plant hydraulic conductance across the entire population, indicating that roots restrict the flow of water to the guard cells and hence limit TR at high VPD. Similarly, Sivasakthi *et al.* (2020) postulated that limited root hydraulic conductance restricted chickpea TR at high VPD (although leaf/stem hydraulic conductance were not measured). Root hydraulic conductance affects the point at which plants reach their maximum TR or begin to reduce TR in response to elevated VPD, allowing plants to maintain higher *g*_s_ and preventing a decline in TR in response to high VPD (Sadok and Sinclair, 2010*b*). In contrast, limited leaf hydraulic conductance restricted transpiration at high VPD in soybean (Sinclair *et al*., 2008; Sadok and Sinclair, 2010*a*, 2012; Devi *et al*., 2016) and peanut (*Arachis hypogaea*) (Devi *et al*., 2012), suggesting variation in the site of hydraulic limitations in these species.

The high genetic variation between the RILs in the transpiration response to VPD and other related traits suggests that the limited transpiration trait at high VPD could be used in crop breeding programmes. QTL analysis identified 13 QTLs associated with the minimum, maximum and BP transpiration, and minimum and maximum whole-plant and root hydraulic conductance traits on chromosomes 1, 3 and 5. Some genomic regions governing frost tolerance and winter hardiness have also been detected on faba bean chromosomes 1 and 3 neighbouring QTLs detected in this study (reviewed in Khazaei *et al*., 2021). Using the same mapping population at the *F*_5_ generation, Khazaei *et al.* (2014) reported QTLs for stomatal morphology and function mainly on chromosome 2. Most of the identified candidate genes in this study were previously reported to regulate plant response to several abiotic stresses (Supplementary Data Table S2). Of these, for instance, the property of qTR_min_1.1 flanking markers were associated with alkaline ceramidase-related genes and 1-aminocyclopropane-1-carboxylate oxidase 3-related genes (Kim *et al*., 1998; Zheng *et al*., 2018; Houben and Van de Poel, 2019). Greater ethylene biosynthesis was associated with stomatal opening of tomato grown at high (90 %) relative humidity (Arve and Torre, 2015). The QTLs qTR_max_1.1 and qTR_max_1.3 were related to oligopeptide transporters (Zhu, 2016; Qi *et al*., 2018; Gong *et al*., 2020) and Basic-Leucine Zipper (BZIP) transcription factor family protein (Yu *et al*., 2020) that are known to play crucial roles in plant responses to several abiotic stresses including water deficit and reactive oxygen species (ROS). Interestingly, the region on chromosome 1 where seven QTLs were co-located (qTR_min_1.2, qK_plant-min_1.1, qK_plant-min_1.2, qK_plant-max_1.1, qK_root-max_1.1, qK_root-min_1.1, and qK_root-min_1.2) accommodated a cytochrome *c* oxidase that when deficient in *Arabidopsis thaliana* lowered sensitivity to ABA (Garcia *et al*., 2016). In wheat (*Triticum aestivum*), six QTLs associated with the transpiration response to VPD were identified, of which one major QTL included genes involved in root hydraulic conductance and ABA signalling (Schoppach *et al*., 2016). Similarly, in soybean, limited transpiration was associated with two QTLs that harbour several candidate genes, including one involved in abiotic stress tolerance (Sarkar *et al*., 2022). In chickpea, transpiration efficiency (ratio of biomass per unit of transpired water) was associated with the ‘*QTL-hotspo*t’ region that harboured four genes associated with drought adaptation (Barmukh *et al*., 2022). These findings confirm the role of genetic populations in detecting genomic regions and candidate genes for water conservation traits under complex genetic control to discriminate the regulation of limited TR at elevated VPD.

## CONCLUSIONS

More than 90 % of the faba bean RILs used in this study restricted TR at high VPD, with considerable variation in the BP at which this occurred. Although genotypes with BPs that restrict TR at high VPD are regarded as suitable for water-deficit environments (Gholipoor *et al*., 2010), in this faba bean population the RILs with a linear TR response to VPD actually had the lowest TR at high VPD. This suggests complex regulation of these transpiration and hydraulic responses to VPD. This study provides the first reports on the genetic control of thre transpiration response to VPD in faba bean. The identified QTLs can be used as potential targets for further genetic studies, and after validation in appropriate germplasm, the linked DNA markers can enable the use of marker-assisted selection to help breed water-conserving faba bean genotypes.

## SUPPLEMENTARY DATA

Supplementary data are available online at https://academic.oup.com/aob and consist of the following. Figure S1: leaf area of RILs derived from Mélodie/2 and ILB 938/2 across the months the plants were harvested. Figure S2: composite interval mapping results for minimum and maximum transpiration rate, whole-plant and root hydraulic conductance, and break-point transpiration. Table S1: mean leaf area of the parental lines and RILs derived from their cross, across time of day and time of year. Table S2: candidate genes associated with QTLs for minimum and maximum transpiration, whole-plant and root hydraulic conductance, and break-point transpiration traits based on BLASTn sequence similarity searches in the *Medicago truncatula* genome.

mcad006_suppl_Supplementary_Figure_S1

mcad006_suppl_Supplementary_Figure_S2

mcad006_suppl_Supplementary_Table_S1

mcad006_suppl_Supplementary_Table_S2

## References

[CIT0001] Abdelmula AA , Link W, Von Kittlitz E, Stelling S. 1999. Heterosis and inheritance of drought tolerance in faba bean, *Vicia faba* L. Plant Breeding 118: 845–849.

[CIT0002] Affortit P , Effa-Effa B, Ndoye MS, et al. 2022. Physiological and genetic control of transpiration efficiency in African rice, *Oryza glaberrima* Steud. Journal of Experimental Botany 73: 5279–5293. doi:10.1093/jxb/erac156. 35429274

[CIT0003] Alghamdi SS , Khan MA, Ammar MH, et al. 2018. Characterization of drought stress-responsive root transcriptome of faba bean (*Vicia faba* L.) using RNA sequencing. 3 Biotech 8: 502. doi:10.1007/s13205-018-1518-2. PMC6258570 30498675

[CIT0004] Arve LE , Torre S. 2015. Ethylene is involved in high air humidity promoted stomatal opening of tomato (*Lycopersicon esculentum*) leaves. Functional Plant Biology 42: 376–386. doi:10.1071/fp14247. 32480682

[CIT0005] Attia Z , Domec JC, Oren R, Way DA, Moshelion M. 2015. Growth and physiological responses of isohydric and isohydric poplars to drought. Journal of Experimental Botany 66: 4373–4381. doi:10.1093/jxb/erv195. 25954045 PMC4493787

[CIT0006] Barmukh R , Roorkiwal M, Dixit GP, et al. 2022. Characterization of ‘*QTL-hotspot*’ introgression lines reveals physiological mechanisms and candidate genes associated with drought adaptation in chickpea. Journal of Experimental Botany: erac348. doi:10.1093/jxb/erac348. PMC9730794 36006832

[CIT0007] Björnsdotter E , Nadzieja M, Chang W, et al. 2021. VC1 catalyses a key step in the biosynthesis of vicine in faba bean. Nature Plants 7: 923–931. doi:10.1038/s41477-021-00950-w. 34226693 PMC7611347

[CIT0008] Buckley TN. 2005. The control of stomata by water balance. New Phytologist 168: 275–292. doi:10.1111/j.1469-8137.2005.01543.x. 16219068

[CIT0009] Bunce JA. 1997. Does transpiration control stomatal responses to water vapor pressure deficit? Plant, Cell & Environment 20: 131–135.

[CIT0010] Carrillo-Perdomo E , Vidal A, Kreplak J, et al. 2020. Development of new genetic resources for faba bean (*Vicia faba* L.) breeding through the discovery of gene-based SNP markers and the construction of a high-density consensus map. Scientific Reports 10: 6790. doi:10.1038/s41598-020-63664-7. 32321933 PMC7176738

[CIT0011] Chongtham SK , Devi EL, Samantara K, et al. 2022. Orphan legumes: harnessing their potential for food, nutritional and health security through genetic approaches. Planta 256: 24. 35767119 10.1007/s00425-022-03923-1

[CIT0012] Choudhary S , Mutava RN, Shekoofa A, Sinclair TR, Prasad PVV. 2013. Is the stay-green trait in sorghum a result of transpiration sensitivity to either soil drying or vapor pressure deficit? Crop Science 53: 2129–2134. doi:10.2135/cropsci2013.01.0043.

[CIT0013] Choudhary S , Sinclair TR, Messina CD, Cooper M. 2014. Hydraulic conductance of maize hybrids differing in transpiration response to vapor pressure deficit. Crop Science 54: 1147–1152. doi:10.2135/cropsci2013.05.0303.

[CIT0014] Collins NC , Tardieu F, Tuberosa R. 2008. Quantitative trait loci and crop performance under abiotic stress: where do we stand? Plant Physiology 147: 469–486. doi:10.1104/pp.108.118117. 18524878 PMC2409033

[CIT0015] Cottage A , Gostkiewicz K, Thomas JE, Borrows R, Torres AM, O’Sullivan DM. 2012. Heterozygosity and diversity analysis using mapped SNPs in a faba bean inbreeding programme. Molecular Breeding 30: 1799–1809. doi:10.1007/s11032-012-9745-4.

[CIT0016] Damour G , Simonneau T, Cochard H, Urban L. 2010. An overview of models of stomatal conductance at the leaf level. Plant, Cell & Environment 33: 1419–1438. doi:10.1111/j.1365-3040.2010.02181.x. 20545879

[CIT0017] Devi MJ , Sadok W, Sinclair TR. 2012. Transpiration response of de-rooted peanut plants to aquaporin inhibitors. Environmental and Experimental Botany 78: 167–172. doi:10.1016/j.envexpbot.2012.01.001.

[CIT0018] Devi MJ , Sinclair TR, Taliercio E. 2016. Silver and zinc inhibitors influence transpiration rate and aquaporin transcript abundance in intact soybean plants. Environmental and Experimental Botany 122: 168–175. doi:10.1016/j.envexpbot.2015.10.006.

[CIT0019] Devi MJ , Sinclair TR, Vadez V. 2010. Genotypic variation in peanut for transpiration response to vapor pressure deficit. Crop Science 50: 191–196. doi:10.2135/cropsci2009.04.0220.

[CIT0020] Dewar RC. 2002. The Ball–Berry–Leuning and Tardieu–Davies stomatal models: synthesis and extension within a spatially aggregated picture of guard cell function. Plant, Cell & Environment 25: 1383–1398.

[CIT0021] Else MA , Davies WJ, Malone M, Jackson MB. 1995. A negative hydraulic message from oxygen-deficient roots of tomato plants? Influence of soil flooding on leaf water potential, leaf expansion, and synchrony between stomatal conductance and root hydraulic conductivity. Plant Physiology 109: 1017–1024. doi:10.1104/pp.109.3.1017. 12228649 PMC161404

[CIT0022] Garcia L , Welchen E, Gey U, Arce AL, Steinebrunner I, Gonzalez DH. 2016. The cytochrome c oxidase biogenesis factor AtCOX17 modulates stress responses in Arabidopsis. Plant, Cell & Environment 39: 628–644. doi:10.1111/pce.12647. 26436309

[CIT0023] Gela TS , Bruce M, Chang W, et al. 2022. Genomic regions associated with chocolate spot (*Botrytis fabae* Sard.) resistance in faba bean (*Vicia faba* L.). Molecular Breeding 42: 35. 10.1007/s11032-022-01307-7 PMC10248645 37312967

[CIT0024] Ghanem ME , Marrou H, Sinclair TR. 2015. Physiological phenotyping of plants for crop improvement. Trends in Plant Science 20: 139–144. doi:10.1016/j.tplants.2014.11.006. 25524213

[CIT0025] Gholipoor M , Prasad PVV, Mutava RN, Sinclair TR. 2010. Genetic variability of transpiration response to vapor pressure deficit among sorghum genotypes. Field Crops Research 119: 85–90.

[CIT0026] Gong Z , Xiong L, Shi H, et al. 2020. Plant abiotic stress response and nutrient use efficiency. Science China Life Sciences 63: 635–674. 32246404 10.1007/s11427-020-1683-x

[CIT0027] Goodstein D , Shu S, Howson R, et al. 2012. Phytozome: a comparative platform for green plant genomics. Nucleic Acids Research 40: D1178–D1186. 22110026 10.1093/nar/gkr944 PMC3245001

[CIT0028] Guiguitant J , Marrou H, Vadez V, et al. 2017. Relevance of limited-transpiration trait for lentil (*Lens culinaris* Medik.) in South Asia. Field Crops Research 209: 96–107.

[CIT0029] Heffelfinger C , Fragoso CA, Lorieux M. 2017. Constructing linkage maps in the genomics era with MapDisto 2.0. Bioinformatics 33: 2224–2225. doi:10.1093/bioinformatics/btx177. 28369214 PMC5870660

[CIT0030] Houben M , Van de Poel B. 2019. 1-Aminocyclopropane-1-carboxylic acid oxidase (ACO): The enzyme that makes the plant hormone ethylene. Frontiers in Plant Science 10: 695. doi:10.3389/fpls.2019.00695. 31191592 PMC6549523

[CIT0031] Jalakas P , Takahashi Y, Waadt R, Schroeder JI, Merilo E. 2021. Molecular mechanisms of stomatal closure in response to rising vapour pressure deficit. New Phytologist 232: 468–475. doi:10.1111/nph.17592. 34197630 PMC8455429

[CIT0032] Jauregui I , Rothwell SA, Taylor SH, Parry MA, Carmo-Silva E, Dodd IC. 2018. Whole plant chamber to examine sensitivity of cereal gas exchange to changes in evaporative demand. Plant Methods 14: 1–13. 30410567 10.1186/s13007-018-0357-9 PMC6211548

[CIT0033] Jayakodi M , Golicz AA, Kreplak J, Fechete LI, Angra D, et al. 2023. The giant diploid faba genome unlocks variation in a global protein crop. Nature (in press). doi:10.1038/s41586-023-05791-5. PMC10033403 36890232

[CIT0034] Kar S , Tanaka R, Korbu LB, et al. 2020. Automated discretization of ‘transpiration restriction to increasing VPD’ features from outdoors high-throughput phenotyping data. Plant Methods 16: 140–160. doi:10.1186/s13007-020-00680-8. 33072176 PMC7565372

[CIT0035] Khan MA , Alghamdi SS, Ammar MH, et al. 2019. Transcriptome profiling of faba bean (*Vicia faba* L.) drought-tolerant variety hassawi-2 under drought stress using RNA sequencing. Electronic Journal of Biotechnology 39: 15–29. doi:10.1016/j.ejbt.2019.02.004.

[CIT0036] Khan HR , Link W, Hocking TJH, Stoddard FL. 2007. Evaluation of physiological traits for improving drought tolerance in faba bean (*Vicia faba* L.). Plant and Soil 292: 205–217.

[CIT0037] Khazaei H , Link W, Street K, Stoddard FL. 2018. ILB 938, a valuable faba bean (*Vicia faba* L.) accession. Plant Genetic Resources 16: 478–482.

[CIT0038] Khazaei H , O’Sullivan DM, Sillanpää MJ, Stoddard FL. 2014. Use of synteny to identify candidate genes underlying QTL controlling stomatal traits in faba bean (*Vicia faba* L.). Theoretical and Applied Genetics 127: 2371–2385. doi:10.1007/s00122-014-2383-y. 25186169

[CIT0039] Khazaei H , O’Sullivan DM, Stoddard FL, et al. 2021. Recent advances in faba bean genetic and genomic tools for crop improvement. Legume Science 3: e75. doi:10.1002/leg3.75. 34977588 PMC8700193

[CIT0040] Khazaei H , Street K, Santanen A, Bari A, Stoddard FL. 2013. Do faba bean (*Vicia faba* L.) accessions from environments with contrasting seasonal moisture availabilities differ in stomatal characteristics and related traits? Genetic Resources and Crop Evolution 60: 2343–2357. doi:10.1007/s10722-013-0002-4.

[CIT0041] Kholová J , Hash CT, Kumar PL, Yadav RS, Kočová M, Vadez V. 2010. Terminal drought-tolerant pearl millet [*Pennisetum glaucum* (L.) R. Br.] have high leaf ABA and limit transpiration at high vapour pressure deficit. Journal of Experimental Botany 61: 1431–1440. doi:10.1093/jxb/erq013. 20142425 PMC2837262

[CIT0042] Kim YS , Choi D, Lee MM, Lee SH, Kim WT. 1998. Biotic and abiotic stress-related expression of 1-aminocyclopropane-1-carboxylate oxidase gene family in *Nicotiana glutinosa* L. Plant & Cell Physiology 6: 565–573. 10.1093/oxfordjournals.pcp.a029406 9697341

[CIT0043] Kosambi DD. 1943. The estimation of map distances from recombination values. Annals of Eugenics 12: 172–175. doi:10.1111/j.1469-1809.1943.tb02321.x.

[CIT0044] Mandour H. 2022. *Genetic variation in transpiration response to evaporative demand in faba bean*. PhD thesis. Lancaster University, Lancaster, UK.

[CIT0045] McAdam SAM , Sussmilch FC, Brodribb TJ. 2016. Stomatal responses to vapour pressure deficit are regulated by high speed gene expression in angiosperms. Plant, Cell & Environment 39: 485–491. doi:10.1111/pce.12633. 26353082

[CIT0046] McAdam SAM , Sussmilch FC, Brodribb TJ, Ross JJ. 2015. Molecular characterization of a mutation affecting abscisic acid biosynthesis and consequently stomatal responses to humidity in an agriculturally important species. AoB Plants 7: plv091. doi:10.1093/aobpla/plv091. 26216469 PMC4583606

[CIT0047] Messina CD , Sinclair TR, Hammer GL, et al. 2015. Limited-transpiration trait may increase maize drought tolerance in the US corn belt. Agronomy Journal 107: 1978–1986. doi:10.2134/agronj15.0016.

[CIT0048] Muktadir MA , Adhikari KN, Merchant A, et al. 2020. Physiological and biochemical basis of faba bean breeding for drought adaptation—a review. Agronomy 10: 1345. doi:10.3390/agronomy10091345.

[CIT0049] O’Sullivan DM , Angra D, Harvie T, Tagkouli V, Warsame A. 2019. A genetic toolbox for *Vicia faba* improvement. In: International conference on legume genetics and genomics, May 13–17, 2019. Dijon, France.

[CIT0050] Qi JS , Song CP, Wang BS, et al. 2018. Reactive oxygen species signaling and stomatal movement in plant responses to drought stress and pathogen attack. Journal of Integrative Plant Biology 60: 805–826. doi:10.1111/jipb.12654. 29660240

[CIT0051] Ryan A , Dodd IC, Rothwell SA, et al. 2016. Gravimetric phenotyping of whole plant transpiration responses to atmospheric vapour pressure deficit identifies genotypic variation in water use efficiency. Plant Science 251: 101–109. 27593468 10.1016/j.plantsci.2016.05.018

[CIT0052] Sadok W , Sinclair TR. 2009. Genetic variability of transpiration response to vapor pressure deficit among soybean (*Glycine max* [L.] Merr.) genotypes selected from a recombinant inbred line population. Field Crops Research 113: 156–160. doi:10.1016/j.fcr.2009.05.002.

[CIT0053] Sadok W , Sinclair TR. 2010a. Transpiration response of ‘slow-wilting’ and commercial soybean (*Glycine max* (L.) Merr.) genotypes to three aquaporin inhibitors. Journal of Experimental Botany 61: 821–829. 19969533 10.1093/jxb/erp350 PMC2814113

[CIT0054] Sadok W , Sinclair TR. 2010b. Genetic variability of transpiration response of soybean *Glycine max* [L.] Merr.) shoots to leaf hydraulic conductance inhibitor AgNO_3_. Crop Science 50: 1423–1430. doi:10.2135/cropsci2009.10.0575.

[CIT0055] Sadok W , Sinclair TR. 2012. Zinc treatment results in transpiration rate decreases that vary among soybean genotypes. Journal of Plant Nutrition 35: 1866–1877. doi:10.1080/01904167.2012.706683.

[CIT0056] Sarkar S , Shekoofa A, McClure A, Gillman JD. 2022. Phenotyping and quantitative trait locus analysis for the limited transpiration trait in an upper-mid south soybean recombinant inbred line population (‘Jackson’ × ‘KS4895’): high throughput aquaporin inhibitor screening. Frontiers in Plant Science 12: 779834. 35126412 10.3389/fpls.2021.779834 PMC8811256

[CIT0057] Schoppach R , Taylor JD, Majerus E, et al. 2016. High resolution mapping of traits related to whole-plant transpiration under increasing evaporative demand in wheat. Journal of Experimental Botany 67: 2847–2860. doi:10.1093/jxb/erw125. 27001921 PMC4861027

[CIT0058] Shekoofa A , Safikhan S, Snider JL, Raper TB, Bourland FM. 2020. Variation in stomatal conductance responses of cotton cultivars to high vapour pressure deficit under controlled and rainfed environments. Journal of Agronomy and Crop Science 207: 332–343. doi:10.1111/jac.12440.

[CIT0059] Sinclair TR , Devi JM, Carter TE. Jr. 2016. Limited-transpiration trait for increased yield for water-limited soybean: from model to phenotype to genotype to cultivars. In: Yin X, Struik PC, eds. Crop systems biology. Berlin: Springer International Publishing, 129–146.

[CIT0060] Sinclair TR , Marrou H, Soltani A, Vadez V, Chandolu KC. 2014. Soybean production potential in Africa. Global Food Security 3: 31–40. doi:10.1016/j.gfs.2013.12.001.

[CIT0061] Sinclair TR , Messina CD, Beatty A, Samples M. 2010. Assessment across the United States of the benefits of altered soybean drought traits. Agronomy Journal 102: 475–482. doi:10.2134/agronj2009.0195.

[CIT0062] Sinclair TR , Zwieniecki MA, Holbrook NM. 2008. Low leaf hydraulic conductance associated with drought tolerance in soybean. Physiologia Plantarum 132: 446–451. doi:10.1111/j.1399-3054.2007.01028.x. 18333998

[CIT0063] Sivasakthi K , Tharanya M, Zaman-Allah M, Kholová J, Thirunalasundari T, Vadez V. 2020. Transpiration difference under high evaporative demand in chickpea (*Cicer arietinum* L.) may be explained by differences in the water transport pathway in the root cylinder. Plant Biology 22: 769–780. 32558986 10.1111/plb.13147

[CIT0064] Skovbjerg CK , Angra D, Robertson-Shersby-Harvie T, et al. 2022. Genetic analysis of global faba bean germplasm maps agronomic traits and identifies strong selection signatures for geographical origin. bioRxiv 2022: 18.500421.

[CIT0065] Sperry JS , Hacke UG, Oren R, Comstock JP. 2002. Water deficits and hydraulic limits to leaf water supply. Plant, Cell & Environment 25: 251–263. doi:10.1046/j.0016-8025.2001.00799.x. 11841668

[CIT0066] Steudle E , Peterson CA. 1998. How does water get through roots? Journal of Experimental Botany 49: 775–788.

[CIT0067] Tamang BG , Monnens D, Anderson JA, Steffenson BJ, Sadok W. 2022. The genetic basis of transpiration sensitivity to vapor pressure deficit in wheat. Physiologia Plantarum 174: e13752. doi:10.1111/ppl.13752. 36281842 PMC9543498

[CIT0068] Taylor J , Butler D. 2017. R package ASMap: efficient genetic linkage map construction and diagnosis. Journal of Statistical Software 79: 1–29.30220889

[CIT0069] Tsuda M , Tyree MT. 2000. Plant hydraulic conductance measured by the high pressure flow meter in crop plants. Journal of Experimental Botany 51: 823–828. 10938875

[CIT0070] Vadez V , Kholova J, Medina S, Kakkera A, Anderberg H. 2014. Transpiration efficiency: New insights into an old story. Journal of Experimental Botany 65: 6141–6153. doi:10.1093/jxb/eru040. 24600020

[CIT0071] Vicente-Serrano SM , Beguería S, Lorenzo-Lacruz J, et al. 2012. Performance of drought indices for ecological, agricultural, and hydrological applications. Earth Interactions 16: 1–27. doi:10.1175/2012ei000434.1.

[CIT0072] Wang S , Basten CJ, Zeng ZB. 2012. Windows QTL Cartographer 25. Raleigh, NC: Department of Statistics, North Carolina State University.

[CIT0073] Wang C , Liu R, Liu Y, et al. 2021. Development and application of the Faba_bean_130K targeted next-generation sequencing SNP genotyping platform based on transcriptome sequencing. Theoretical and Applied Genetics 134: 3195–3207. doi:10.1007/s00122-021-03885-0. 34117907

[CIT0074] Weatherley PE. 1982. Water uptake and flow into roots. In: Lange OL, Nobel PS, Osmond CB, Ziegler H, eds. Encyclopaedia of plant physiology. Berlin: Springer, 12: 79–109.

[CIT0075] Webb A , Cottage A, Wood T, et al. 2016. A SNP-based consensus genetic map for synteny-based trait targeting in faba bean (*Vicia faba* L.). Plant Biotechnology Journal 14: 177–185. 25865502 10.1111/pbi.12371 PMC4973813

[CIT0076] Wu X , Fan Y, Li L, Liu Y. 2020. The influence of soil drought stress on the leaf transcriptome of faba bean (*Vicia faba* L.) in the Qinghai–Tibet plateau. 3 Biotech 10: 381. doi:10.1007/s13205-020-02374-3. PMC7413945 32802723

[CIT0077] Würschum T. 2012. Mapping QTL for agronomic traits in breeding populations. Theoretical and Applied Genetics 125: 201–210. doi:10.1007/s00122-012-1887-6. 22614179

[CIT0078] Yan J , Yang X, Shah T, et al. 2010. High-throughput SNP genotyping with the Golden Gate assay in maize. Molecular Breeding 25: 441–451.

[CIT0079] Yu Y , Qian Y, Jiang M, et al. 2020. Regulation mechanisms of plant basic leucine zippers to various abiotic stresses. Frontiers in Plant Science 11: 1258. doi:10.3389/fpls.2020.01258. 32973828 PMC7468500

[CIT0080] Zheng P , Wu JX, Sahu SK, et al. 2018. Loss of alkaline ceramidase inhibits autophagy in Arabidopsis and plays an important role during environmental stress response. Plant, Cell & Environment 41: 837–849. doi:10.1111/pce.13148. 29341143

[CIT0081] Zhu J-K. 2016. Abiotic stress signaling and responses in plants. Cell 167: 313–324. doi:10.1016/j.cell.2016.08.029. 27716505 PMC5104190

