# Supplementary figures and images for: Identifying physiological and genetic determinants of faba bean transpiration response to evaporative demand
Source: Ann Bot. 2023 Jan 19;131(3):533–44. doi: 10.1093/aob/mcad006 (PMC10072112; doi:10.1093/aob/mcad006)

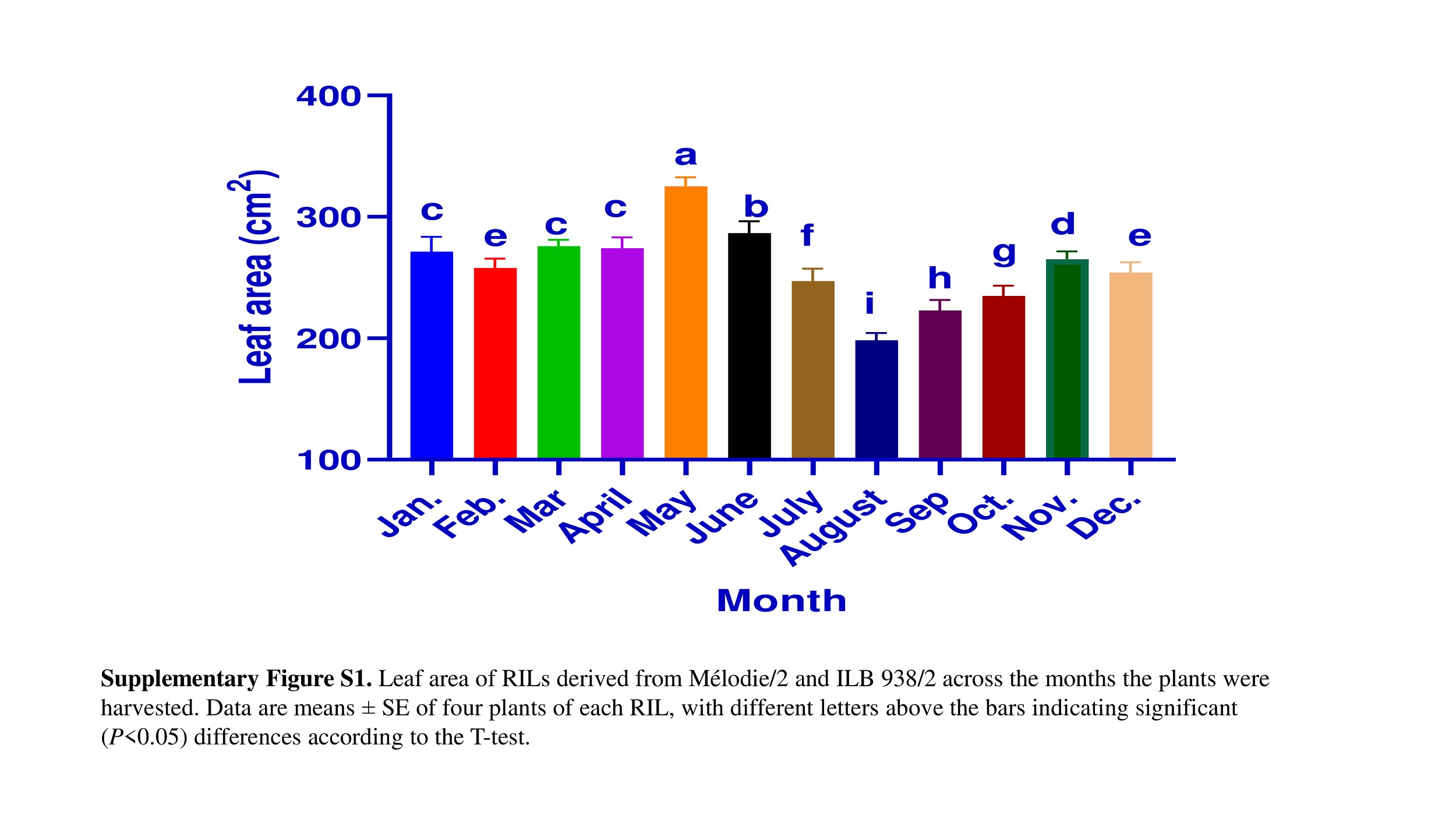

Supplement: mcad006_suppl_Supplementary_Figure_S1 [file mcad006_suppl_supplementary_figure_s1.jpeg]

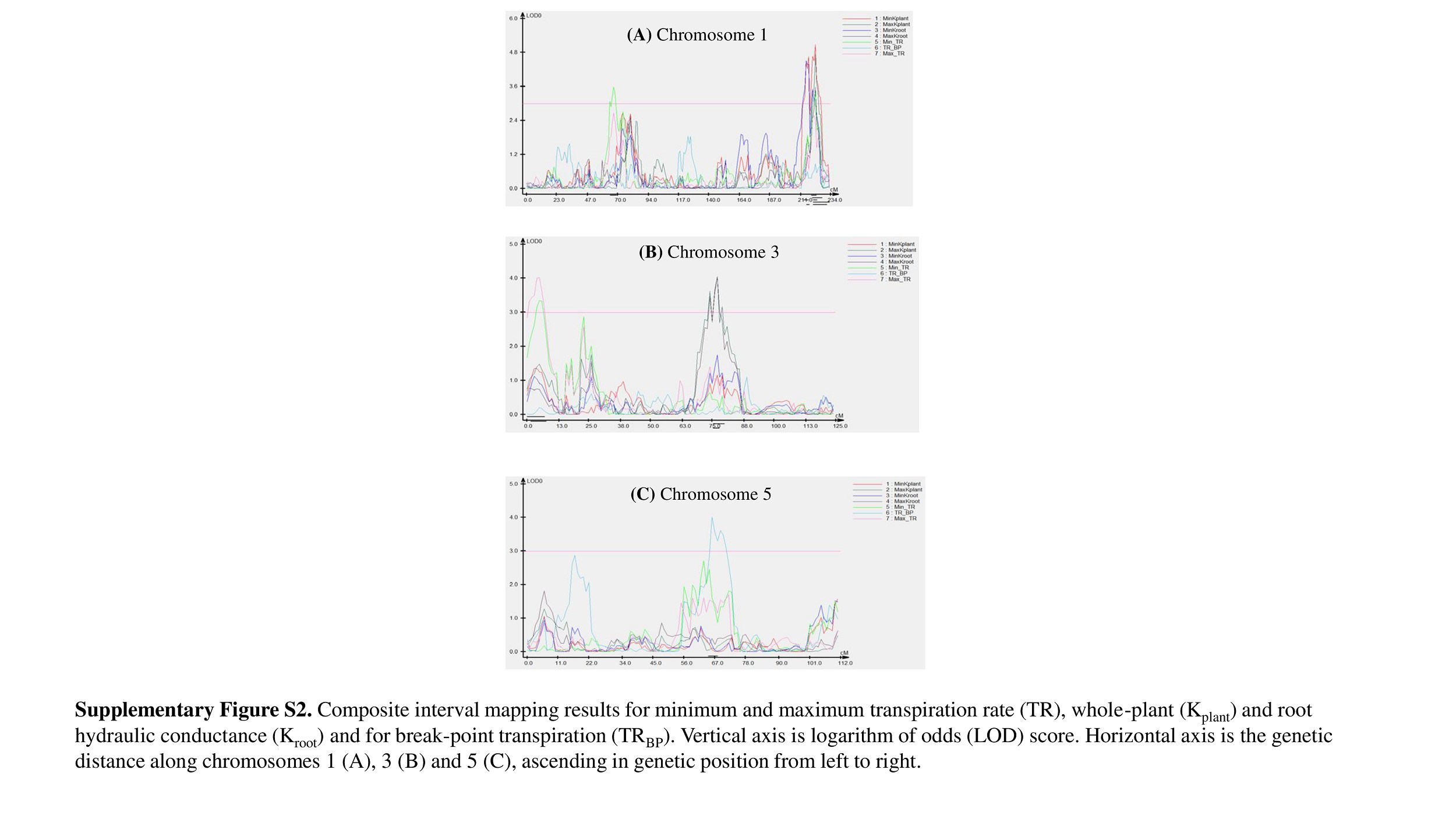

Supplement: mcad006_suppl_Supplementary_Figure_S2 [file mcad006_suppl_supplementary_figure_s2.jpeg]
