# Supplementary material for: Identifying physiological and genetic determinants of faba bean transpiration response to evaporative demand
Source: Ann Bot. 2023 Jan 19;131(3):533–44. doi: 10.1093/aob/mcad006 (PMC10072112; doi:10.1093/aob/mcad006)
Supplement: mcad006_suppl_Supplementary_Table_S1 [file mcad006_suppl_supplementary_table_s1.docx]

**Supplementary Table S1.** Mean of leaf area of the parental lines and RILs derived from their cross, across time of day and time of year. Data are means ± SE of 3-4 plants of each genotype. *P*-values from ANOVA for the genotypic differences between the RILs for leaf area across months the plants were planted provided at the end of the table.

| Parent/RIL | Leaf area (cm^2^) ± SE |
| --- | --- |
| Mélodie/2 | 235 ± 1.50 (n= 4) |
| ILB 938/2 | 215 ± 4.37 (n= 4) |
| L141 | 211 ± 1.11 (n= 4) |
| L86 | 226 ± 4.47 (n= 4) |
| M49 | 208 ± 3.28 (n= 4) |
| H103 | 250 ± 5.92 (n= 4) |
| H45 | 262 ± 2.44 (n= 3) |
| H197 | 205 ± 2.01 (n= 3) |
| H7 | 248 ± 3.33 (n= 3) |
| H200 | 225 ± 2.25 (n= 3) |
| H134 | 210 ± 3.44 (n= 3) |
| H142 | 259 ± 5.22 (n=4) |
| H37 | 205 ± 3.25 (n= 3) |
| L114 | 234 ± 2.27 (n= 4) |
| H205 | 206 ± 2.21 (n= 3) |
| L90 | 200 ± 2.01 (n= 4) |
| L198 | 224 ± 1.55 (n= 3) |
| L175 | 199 ± 1.79 (n= 3) |
| L126 | 212 ± 1.52 (n= 3) |
| H196 | 265 ± 4.55 (n= 4) |
| H8 | 216 ± 2.44 (n= 3) |
| L104 | 267 ± 2.80 (n= 3) |
| H28 | 240 ± 4.25 (n= 3) |
| L9 | 227 ± 1.63 (n= 3) |
| L168 | 234 ± 2.80 (n= 3) |
| H98 | 234 ± 2.14 (n= 4) |
| L174 | 257 ± 2.24 (n= 3) |
| H82 | 201 ± 2.75 (n= 3) |
| L78 | 197 ± 1.17 (n= 4) |
| L91 | 246 ± 1.97 (n= 3) |
| H54 | 215 ± 2.15 (n= 4) |
| H73 | 269 ± 3.67 (n= 3) |
| L179 | 253 ± 1.50 (n= 4) |
| H63 | 196 ± 0.87 (n= 4) |
| L57 | 222 ± 2.53 (n= 4) |
| H158 | 215 ± 2.11 (n= 4) |
| H188 | 240 ± 2.24 (n=4) |
| H88 | 214 ± 4.83 (n= 4) |
| H64 | 245 ± 1.76 (n= 4) |
| H209 | 269 ± 2.85 (n= 3) |
| H22 | 223 ± 4.26 (n= 4) |
| H30 | 253 ± 6.88 (n=3) |
| H145 | 238 ± 2.02 (n=3) |
| L48 | 243 ± 2.85 (n= 3) |
| L191 | 207 ± 4.36 (n= 3) |
| M31 | 272 ± 4.98 (n= 3) |
| L160 | 217 ± 3.99 (n= 3) |
| L184 | 210 ± 4.99 (n= 3) |
| H206 | 211 ± 4.71 (n= 4) |
| L93 | 219 ± 1.42 (n= 4) |
| H42 | 208 ± 3.52 (n= 4) |
| H53 | 210 ± 3.47 (n= 3) |
| L94 | 209 ± 1.63 (n= 3) |
| H173 | 261± 4.27 (n= 4) |
| L151 | 242 ± 3.97 (n= 4) |
| L1 | 272 ± 3.53 (n= 4) |
| L87 | 272 ± 6.75 (n= 3) |
| L41 | 201 ± 1.18 (n= 4) |
| H177 | 228 ± 4.94 (n= 4) |
| L68 | 241 ± 4.94 (n= 3) |
| H149 | 232 ± 2.75 (n= 4) |
| H47 | 208 ± 2.12 (n= 4) |
| H46 | 203 ± 0.82 (n= 4) |
| L165 | 209 ± 1.42 (n= 3) |
| H131 | 205 ± 1.63 (n= 4) |
| H144 | 215 ± 1.93 (n= 4) |
| L208 | 214 ± 5.25 (n= 3) |
| L199 | 204 ± 3.27 (n=3) |
| H194 | 280 ± 2.70 (n= 4) |
| H14 | 223 ± 2.24 (n= 4) |
| H38 | 237± 1.02 (n= 3) |
| L89 | 228 ± 1.11 (n= 4) |
| H170 | 239 ± 1.38 (n= 3) |
| L147 | 215± 2.77 (n= 4) |
| L135 | 231 ± 2.96 (n=3) |
| L2 | 218 ± 2.88 (n=3) |
| L12 | 238 ± 5.50 (n= 4) |
| L36 | 210 ± 4.18 (n= 3) |
| L74 | 216 ± 2.91 (n=3) |
| H146 | 254 ± 3.12 (n= 4) |
| H69 | 227 ± 4.16 (n= 4) |
| H172 | 222 ± 3.53 (n= 4) |
| L34 | 230 ± 1.17 (n= 4) |
| H62 | 253 ± 5.18 (n= 4) |
| H27 | 272 ± 3.11 (n= 4) |
| H99 | 228 ± 2.0 (n=4) |
| H112 | 256 ± 3.32 (n= 4) |
| H124 | 201± 2.04 (n= 3) |
| L105 | 207 ± 1.75 (n= 3) |
| L11 | 236 ± 2.04 (n= 3) |
| H137 | 253 ± 3.32 (n= 4) |
| L6 | 232 ± 3.67 (n= 3) |
| L193 | 222 ± 2.51 (n= 3) |
| L159 | 179 ± 1.17 (n= 3) |
| L168 | 234 ± 3.12 (n= 4) |
| H109 | 216 ± 2.04 (n= 3) |
| L128 | 203 ± 1.42 (n= 3) |
| H7 | 248 ± 2.12 (n= 4) |
| H3 | 246 ± 1.89 (n= 4) |
| L10 | 232 ± 2.06 (n= 4) |
| H21 | 258 ± 4.55 (n= 4) |
| L17 | 232 ± 46.93 (n= 3) |
| L34 | 231± 1.17 (n= 4) |
| H102 | 204 ± 4.18 (n= 4) |
| H108 | 203 ± 1.84 (n= 4) |
| L133 | 212 ± 4.46 (n= 4) |
| H182 | 211± 4.71 (n=4) |
| H18 | 237 ± 4.22 (n= 4) |
| H100 | 208 ± 5.25 (n= 4) |
| H59 | 223 ± 2.92 (n= 4) |
| H117 | 200 ± 2.69 (n= 4) |
| H167 | 211 ± 2.47 (n= 4) |
| H92 | 225 ± 3.29 (n= 4) |
| L136 | 216 ± 1.17 (n= 4) |
| H33 | 205 ± 4.89 (n= 4) |
| H201 | 250 ± 3.88 (n= 4) |
| L152 | 238 ± 2.94 (n= 4) |
| H180 | 240 ± 3.26 (n= 3) |
| L185 | 237± 3.72 (n= 4) |
| H190 | 227 ± 2.22 (n= 4) |
| L124 | 212 ± 6.25 (n=3) |
| H161 | 208 ± 0.85 (n= 3) |
| L129 | 207 ± 4.75 (n= 3) |
| H101 | 243 ± 4.30 (n= 4) |
| H210 | 239 ± 3.94 (n= 3) |
| H189 | 224 ± 3.94 (n= 3) |
| H147 | 225 ± 4.83 (n= 4) |
| L153 | 217 ± 1.92 (n= 3) |
| H81 | 224 ± 3.60 (n=3) |
| L25 | 211 ± 5.91 (n= 4) |
| L24 | 247 ± 3.50 (n= 4) |
| H75 | 226 ± 3.53 (n= 4) |
| H69 | 227 ± 3.12 (n= 4) |
| L43 | 205 ± 0.58 (n= 4) |
| H29 | 224 ± 0.47 (n= 4) |
| H70 | 248 ± 1.86 (n= 4) |
| L77 | 247 ± 3.50 (n= 3) |
| L211 | 253 ± 2.0 (n= 3) |
| L124 | 213 ± 1.99 (n= 3) |
| H178 | 210 ± 3.47 (n= 3) |
| H187 | 209 ± 2.71 (n= 4) |
| L176 | 217 ± 1.99 (n= 3) |
| L118 | 267 ± 3.84 (n= 4) |
| L79 | 227 ± 2.69 (n= 4) |
| L163 | 209 ± 3.94 (n= 3) |
| H55 | 254 ± 1.99 (n= 4) |
| H32 | 219 ± 3.66 (n= 4) |
| L20 | 243 ± 4.25 (n= 4) |
| H157 | 213 ± 2.44 (n= 3) |
| M51 | 211 ± 4.25 (n= 3) |
| L113 | 220 ± 1.32 (n=3) |
| H83 | 234 ± 3.04 (n= 3) |
| H111 | 244 ± 3.08 (n=4) |
| H27 | 258 ± 2.54 (n= 4) |
| L6 | 232 ± 2.02 (n= 3) |
| H203 | 234 ± 3.47 (n= 3) |
| L65 | 271 ± 2.26 (n= 3) |
| H26 | 235 ± 2.04 (n= 3) |
| H110 | 224 ± 2.24 (n= 3) |
| H112 | 200 ± 1.02 (n= 3) |
| L115 | 200 ± 1.50 (n= 4) |
| H60 | 261 ± 5.78 (n= 3) |
| L123 | 277 ± 2.78 (n= 3) |
| H148 | 207 ± 2.24 (n= 4) |
| H139 | 220 ± 5.19 (n= 4) |
| H66 | 261 ± 4.73 (n= 4) |
| L61 | 235 ± 3.26 (n= 4) |
| H84 | 284 ± 8.17 (n= 4) |
| L125 | 212 ± 2.61 (n= 4) |
| H154 | 219 ± 2.29 (n= 3) |
| H4 | 253 ± 2.65 (n= 3) |
| H155 | 206 ± 1.42 (n= 3) |
| H207 | 219 ± 2.26 (n= 3) |
| L107 | 230 ± 4.75 (n= 3) |
| H58 | 246 ± 23.80 (n= 4) |
| L121 | 258 ± 1.92 (n= 3) |
| L192 | 226 ± 1.33 (n=3) |
|  |  |
| ANOVA | *P*-value |
| RIL | 0.500 |
| Month | *<0.001* |
| Genotype × Month | 0.700 |
