# Supplementary material for: Identifying physiological and genetic determinants of faba bean transpiration response to evaporative demand
Source: Ann Bot. 2023 Jan 19;131(3):533–44. doi: 10.1093/aob/mcad006 (PMC10072112; doi:10.1093/aob/mcad006)
Supplement: mcad006_suppl_Supplementary_Table_S2 [file mcad006_suppl_supplementary_table_s2.docx]

**Supplementary Table S2.** Candidate genes associated with QTLs for minimum and maximum transpiration, whole-plant and root hydraulic conductance and break-point transpiration traits based on BLASTn sequence similarity searches in the *Medicago truncatula* (Mt4.0v1) genome. The genes were queried using sequences of the SNP markers identified in the interval between the two stable QTLs.

| **QTL** | **Gene ID#** | **Position#** | **e -value** | **Descriptions** |
| --- | --- | --- | --- | --- |
| **qTR_min_1.1**  **qTR_min_3.1** | Medtr2g025120  Medtr4g132765  Medtr1g022365 | chr2:8925944..8928216  chr4:55522668..55527302  chr1:7086220..7088202 | 1.234e-19  4.01e-13  3.76e-7 | OXIDOREDUCTASE, 2OG-FE /OXYGENASE FAMILY PROTEIN  PTHR33472:SF3 -OXIDOREDUCTASE.  Co-expressed with genes in roots specific co-expression subnetwork. |
| **qTR_min_1.1**  **qTR_min_3.1** | Medtr2g025540  Medtr4g133000  Medtr1g021670 | chr2:9128940..9133614  chr4:55643714..55648105  chr1:6571053..6574863 | 1.82e-17  7.25e-10  4.89e-12 | PTHR12956//PTHR12956:SF17 & SF28 ALKALINE CERAMIDASE-RELATED.Co-expressed with genes in leaf-specific co-expression subnetwork. |
| **qTR_min_1.1** | Medtr2g025180 | chr2:8956289..8961013 | 1.71e-11 | PTHR11216//PTHR11216:SF76 - EH DOMAIN. Co-expressed with genes in roots specific co-expression subnetwork. |
| **qTR_min_1.1** | Medtr2g025710 | chr2:9201966..9203846 | 4.89e-12 | Long-chain-alcohol O-fatty-acyltransferase / Wax synthase.  Co-expressed with genes in roots. specific co-expression subnetwork |
| **qTR_min_1.1** | Medtr3g083370  Medtr5g085330 | chr3:37628014..37630303  chr5:36870741..36872929 | 7.25e-10  1.71e-11 | PTHR10209:SF193 –  1-AMINOCYCLOPROPANE-1-CARBOXYLATE OXIDASE 3-RELATED.  Co-expressed with genes in leaf specific co-expression subnetwork. |
| **qTR_min_1.2**  **qK_plant-min_1.1**  **qK_root-min_1.1** | Medtr3g115220 | chr3:53861604..53869489 | 2.53e-9 | PTHR31818:SF1 – O FUCOSYLTRANSFERASE-LIKE PROTEIN. Co-expressed with genes in leaf specific co-expression subnetwork. |
| **qK_plant-min_1.2**  **qK_plant-max_1.1**  **qK_root-min_1.1**  **qK_plant-max_1.2** | Medtr5g097010 | chr5:42463028..42466972 | 9.43e-15 | PTHR22572:SF101 - GLUCOSE-1-PHOSPHATE ADENYL TRANSFERASE SMALL SUBUNIT, CHLOROPLASTIC  Co-expressed with genes in leaf specific co-expression subnetwork. |
| **qK_plant-min_1.2**  **qK_plant-max_1.1**  **qK_root-min_1.1**  **qK_plant-max_1.2** | Medtr5g096890  Medtr3g061720 | chr5:42397183..42404142  chr3:24621565..24627200 | 3.52e-20  5.95e-11 | PTHR12670:SF4 - NEUTRAL CERAMIDASE-RELATED.  Co-expressed with genes in leaf specific co-expression subnetwork. |
| **qK_plant-min_1.2**  **qK_plant-max_1.1**  **q_Kroot-min_1.1**  **qK_plant-max_1.2** | Medtr5g096660 | chr5:42271151..42278841 | 1.31e-25 | PF00485//PF14681 –Phosphoribulo-kinase / Uridine kinase family (PRK) // Uracil phosphoribosyltransferase (UPRTase). Co-expressed with genes in leaf specific co-expression. subnetwork |
| **qK_plant-min_1.2**  **qK_plant-max_1.1**  **qK_root-min_1.1**  **qK_plant-max_1.2** | Medtr7g029450 | chr7:10536308..10543432 | 4.89-12 | PTHR10285:SF71 - URIDINE KINASE-LIKE PROTEIN 1, CHLOROPLASTIC-RELATED. Co-expressed with genes in leaf specific co-expression subnetwork. |
| **qK_plant-min_1.2**  **qK_plant-max_1.1**  **qK_root-min_1.1**  **qK_plant-max_1.2** | Medtr3g061030 | chr3:24230200..24237140 | 5.95e-11 | 2.4.2.9//2.7.1.48 - Uracil phosphoribosyl-transferase / UMP pyrophosphorylase // Uridine kinase / Uridine monophosphokinase.  Co-expressed with genes in nodules specific co-expression subnetwork. |
| **qK_plant-min_1.2**  **qK_plant-max_1.1**  **qK_root-min_1.1**  **qK_plant-max_1,2** | Medtr5g096670 | chr5:42278942..42281440 | 5.27e-24 | (PTHR11627:SF19 -FRUCTOSE-BISPHOSPHATE ALDOLASE-RELATED  Exhibits leaf specific expression. |
| **qK_plant-min_1.2**  **qK_plant-max_1.1**  **qK_root-min_1.1**  **qK_plant-max_1.2** | Medtr5g096830  Medtr3g095790 | chr5:42354421..42358871  chr3:43769207..43772320 | 2.08e-10  3.76e-7 | PTHR23289 - CYTOCHROME C OXIDASE ASSEMBLY PROTEIN COX15. Co-expressed with genes in nodules specific co-expression subnetwork. Co-expressed with genes in leaf specific co-expression subnetwork. |
| **qK_plant-min_1.1**  **qK_plant-max_1.1**  **qK_root-min_1.2**  **qK_plant-max_1.2** | Medtr5g096970 | chr5:42438617..42447850 | 6.79e-23 | PTHR11902:SF13 - CYTOSOLIC ENOLASE 3. Co-expressed with genes in leaf specific co-expression subnetwork |
| **qK_root-max_1.1** | Medtr5g094770 | chr5:41420060..41421993 | 1.5e-18 | PF01535//PF13041//PF13812 - PPR repeat (PPR) // PPR repeat family (PPR_2) // Pentatricopeptide repeat domain (PPR_3). |
| **qK_root-min_1.1** | Medtr1g018480 | chr1:5334172..5343203 | 3.08e-8 | PTHR11216:SF72 - CALCIUM-BINDING EF HAND-CONTAINING PROTEIN.  Co-expressed with genes in leaf specific co-expression subnetwork |
| **qK_root-min_1.1** | Medtr5g099240 | chr5:43501965..43509933 | 1.5e-18 | PTHR24349:SF83 - CALCIUM-DEPENDENT PROTEIN KINASE 6.  Co-expressed with genes in roots specific co-expression subnetwork. |
| **qK_plant--max_1.1** | Medtr5g095470 | chr5:41729018..41729984 | 1.4e-12 | PTHR23416:SF56 - SERINE ACETYLTRANSFERASE 1, CHLOROPLASTIC-RELATED. |
| **qTR_min_1.2**  **qK_plant-min_1.1** | Medtr4g100810 | chr4:41599236..41604242 | 2.37e-3 | PF10250 - GDP-fucose protein O-fucosyl-transferase (O-FucT). |
| **qTR_min_1.2**  **qK_plant-min_1.1**  **qTR_min_2.1** | Medtr5g098940  Medtr5g087820 | chr5:43313469..43316302  chr5:38090611..38094429 | 1.31e-25  7.74e-16 | PTHR11753//PTHR11753:SF16 - CLATHRIN COAT ASSEMBLY PROTEIN.  Co-expressed with genes in leaf specific co-expression subnetwork. |
| **qTR_min_1.2**  **qK_plant-min_1.1** | Medtr5g099010 | chr5:43363456..43367269 | 9.43e-15 | PTHR31818:SF0 - PROTEIN ROOT HAIR SPECIFIC 17. |
| **qTR_max_3.1** | Medtr1g026540 | chr1:8655619..8658952 | 2.89e-21 | PTHR10366//PTHR10366:SF355 - NAD DEPENDENT EPIMERASE/DEHYDRATASE  Co-expressed with genes in leaf specific co-expression subnetwork  Oxidoreductase activity |
| **qTR_max_3.1** | Medtr2g087640 | chr2:36868869..36873363 | 1.5e-18 | PTHR19877 - WD40 REPEAT PROTEIN. |
| **qTR_max_3.1** | Medtr1g024005 | chr1:7759631..7761462 | 2.53e-9 | PTHR10108//PTHR10108:SF837 – METHYLTRANSFERASE.  Co-expressed with genes in leaf specific co-expression subnetwork. |
| **qTR_max_3.1** | Medtr1g023690 | chr1:7618000..7621776 | 3.52e-20 | PTHR13690:SF76 - BASIC-LEUCINE ZIPPER (BZIP) TRANSCRIPTION FACTOR FAMILY PROTEIN. |
| **qTR_max_3.1** | Medtr4g019450 | chr4:6083889..6090270 | 1.71e-11 | PTHR11850:SF82 - BEL1-LIKE HOMEODOMAIN PROTEIN 8-RELATED.  Co-expressed with genes in leaf specific co-expression subnetwork. |
| **qTR_max_3.1** | Medtr1g019870 | chr1:6062058..6066368 | 4.89e-12 | KOG2659 - LisH motif-containing protein. Co-expressed with genes in roots specific co-expression subnetwork. |
| **qTR_min_1.3**  **qTR_max_3.1** | Medtr1g022360 | chr1:7083958..7084997 | 6.79e-23 | PF01535 - PPR repeat (PPR)  Co-expressed with genes in leaf specific co-expression subnetwork. |
| **qTR_min_3.1**  **qTR_max_3.1** | Medtr3g112020 | chr3:52412285..52417563 | 2.08e-10 | PTHR13061//PTHR13061:SF10 - DYNACTIN SUBUNIT P25.  Co-expressed with genes in roots specific co-expression subnetwork. |
| **qTR_min_3.1**  **qTR_max_3.1** | Medtr1g022225 | chr1:6955941..6957574 | 1.5e-18 | PTHR13930 - RSAFD1-RELATED.  Co-expressed with genes in roots specific co-expression subnetwork. |
| **qTR_min_3.1**  **qTR_max_1.3** | Medtr1g022190 | chr1:6934778..6938822 | 4.01e-18 | PTHR10996:SF123 - ERYTHRONATE-4-PHOSPHATE DEHYDROGENASE FAMILY PROTEIN.  Co-expressed with genes in leaf specific co-expression subnetwork |
| **qTR_min_3.1**  **qTR_max_3.1** | Medtr1g022160 | chr1:6892246..6896988 | 2.08e-10 | PTHR32227:SF16 - GLUCAN ENDO-1,3-BETA-GLUCOSIDASE 7-RELATED.  Co-expressed with genes in roots specific co-expression subnetwork. |
| **qTR_min_3.1**  **qTR_max_3.1** | Medtr1g021925 | chr1:6671114..6675091 | 1.82e-17 | PTHR10983:SF25 - 1-ACYL-SN-GLYCEROL-3-PHOSPHATE ACYLTRANSFERASE 4-RELATED.  Co-expressed with genes in leaf specific co-expression subnetwork |
| **qTR_min_3.1**  **qTR_max_3.1** | Medtr1g021895 | chr1:6658323..6660432 | 1.82e-17 | PTHR12313//PTHR12313:SF10 - RNF5. Co-expressed with genes in roots specific co-expression subnetwork. |
| **qTR_min_3.1**  **qTR_max_3.1** | Medtr1g021950 | chr1:6680254..6688043 | 9.43e-15 | Leishmanolysin / Promastigote surface endopeptidase.  Co-expressed with genes in roots specific co-expression subnetwork. |
| **qTR_min_3.1**  **qTR_max_3.1** | Medtr3g111530 | chr3:52111957..52113811 | 2.89e-2 | 4.2.1.92 – Hydroperoxide dehydratase / Hydroperoxide isomerase  Co-expressed with genes in leaf. specific co-expression subnetwork |
| **qK_plant-max_3.1**  **qK_root-max_3.1** | Medtr1g032520 | chr1:11570552..11577948 | 4.58e-6 | PF07058 - Microtubule-associated protein 70 (MAP70).  Co-expressed with genes in nodules specific co-expression subnetwork |
| **qK_root-max_3.1** | Medtr8g105260 | chr8:44396772..44400394 | 6.79e-4 | KOG0508//KOG4412 - Ankyrin repeat protein // 26S proteasome regulatory complex, subunit PSMD10. Co-expressed with genes in leaf specific co-expression subnetwork. |
| **qTR_BP_5.1** | Medtr1g059700  Medtr7g105050 | chr1:25950770..25955589  chr7:42588771..42592563 | 7.74e-16  7.74e-16 | 3.2.1.26 - Beta-fructofuranosidase / Saccharase |
| **qTR_BP_5.1** | Medtr4g099100 | chr4:41027737..41033129 | 2.89e-2 | PTHR12439 - PLACENTAL PROTEIN 11-RELATED |
| **qTR_BP_5.1** | Medtr4g052940 | chr4:19205590..19210680 | 1.31e-6 | KOG0110//KOG0131//KOG0148 - RNA-binding protein (RRM superfamily) // Splicing factor 3b, subunit 4 // Apoptosis-promoting RNA-binding protein TIA-1/TIAR (RRM superfamily). |
| **qTR_BP_5.1** | Medtr7g105790 | chr7:42945241..42946436 | 5.95e-11 | PTHR33057:SF17 - GB  Co-expressed with genes in leaf specific co-expression subnetwork. |
| **qTR_BP_5.1** | Medtr7g105800 | chr7:42948167..42955987 | 7.25e-10 | PTHR13390 – LIPASE co-expressed with genes in nodules specific co-expression subnetwork. |
| **qTR_BP_5.1** | Medtr7g105870  Medtr7g106010  Medtr7g106000 | chr7:42989208..42990385  chr7:43054682..43055402  chr7:43048977..43049967 | 2.53e-9  5.58e-5  2.37e-3 | PTHR31415:SF4 - HARPIN-INDUCED PROTEIN-LIKE-RELATED.  Co-expressed with genes in roots specific co-expression subnetwork |
| **qTR_BP_5.1** | Medtr7g105100 | chr7:42613545..42617300 | 4.01e-13 | PTHR10992//PTHR10992:SF691 - ALPHA/BETA HYDROLASE FOLD-CONTAINING PROTEIN  Co-expressed with genes in leaf specific co-expression subnetwork. |
| **qTR_BP_5.1** | Medtr7g105170 | chr7:42639030..42640463 | 1.08e-7 | PF02365 - No apical meristem (NAM) protein (NAM). |
| **qTR_BP_5.1** | Medtr7g105030 | chr7:42575595..42582054 | 2.39e-11 | PTHR24115:SF536 - 125 KDA KINESIN-RELATED PROTEIN-RELATED. Co-expressed with genes in roots specific co-expression subnetwork. |
| **qTR_BP_5.1** | Medtr7g104890 | chr7:42517178..42524740 | 1.5e-18 | PTHR11564//PTHR11564:SF19 - GTPASE CONTAINING FAMILY OF SIGNAL RECOGNITION PARTICLE PROTEINS.  Co-expressed with genes in leaf specific co-expression subnetwork. |
| **qTR_BP_5.1** | Medtr7g104800 | chr7:42483009..42489150 | 2.89e-21 | KOG0117 - Heterogeneous nuclear ribonucleoprotein R (RRM superfamily).  Co-expressed with genes in roots specific co-expression subnetwork. |
| **qTR_BP_5.1** | Medtr7g010360 | chr7:2533492..2542856 | 2.37e-3 | KOG0123 - Polyadenylate-binding protein (RRM superfamily).  Co-expressed with genes in leaf specific co-expression subnetwork. |
| **qTR_max_1.1** | Medtr2g011480 | chr2:2789349..2793998 | 3.52e-20 | PTHR13063 - ENOS INTERACTING PROTEIN. Co-expressed with genes in nodules specific co-expression subnetwork. |
| **qTR_max_1.1** | Medtr2g009980  Medtr7g092250  Medtr3g111350 | chr2:2169922..2176627 (PAC:31067297)  chr7:36534179..36538356  chr3:52030513..52035160 | 6.79e-23  5.58e-5  2.53e-9 | (PTHR22601: SF8, SF11, SF23 - OLIGOPEPTIDE TRANSPORTER 1,2,4.  Co-expressed with genes in leaf specific co-expression subnetwork. |
| **qTR_max_1.1** | Medtr3g080870 | chr3:36602875..36608789 | 1.31e-6 | PTHR22601//PTHR22601:SF12 - ISP4 LIKE PROTEIN  Component of root urea treatment specific co-expression subnetwork |
